# Gut Microbiota Serves as a Crucial Independent Biomarker in Inflammatory Bowel Disease (IBD)

**DOI:** 10.3390/ijms26062503

**Published:** 2025-03-11

**Authors:** Bharti Sharma, George Agriantonis, Kate Twelker, Danielle Ebelle, Samantha Kiernan, Maham Siddiqui, Aditi Soni, Sittha Cheerasarn, Whenzdjyny Simon, Winston Jiang, Angie Cardona, Jessica Chapelet, Alexandra Z. Agathis, Alejandro Gamboa, Jasmine Dave, Juan Mestre, Navin D. Bhatia, Zahra Shaefee, Jennifer Whittington

**Affiliations:** 1 Department of Surgery, NYC Health and Hospitals—Elmhurst, New York, NY 11373, USA; sharmab7@nychhc.org (B.S.); agriantg@nychhc.org (G.A.); kiernans1@nychhc.org (S.K.); cheerass@nychhc.org (S.C.); acardona@sgu.edu (A.C.); davej@nychhc.org (J.D.); mestreju@nychhc.org (J.M.); bhatian1@nychhc.org (N.D.B.); shafaeez1@nychhc.org (Z.S.); 2 Department of Surgery, Icahn School of Medicine at Mount Sinai, New York, NY 10029, USA; winston.jiang@mountsinai.org (W.J.); alexandra.agathis2@mountsinai.org (A.Z.A.); 3 Department of Medicine, St. George’s University, Grenada FZ818, West Indies; debelle@sgu.edu (D.E.); msiddiq8@sgu.edu (M.S.); djynysimon@gmail.com (W.S.); jchapele@sgu.edu (J.C.); 4 Department of Medicine, Medical University of the Americas, Devens, MA 01434, USA; a.gamboa@mua.edu

**Keywords:** inflammatory bowel disease, gut microbiota, Crohn’s disease, ulcerative colitis, glycosylation, morphogen, podoplanin

## Abstract

Inflammatory bowel disease (IBD), encompassing Crohn’s disease (CD), ulcerative colitis (UC), and IBD unclassified (IBD-U), is a complex intestinal disorder influenced by genetic, environmental, and microbial factors. Recent evidence highlights the gut microbiota as a pivotal biomarker and modulator in IBD pathogenesis. Dysbiosis, characterized by reduced microbial diversity and altered composition, is a hallmark of IBD. A consistent decrease in anti-inflammatory bacteria, such as *Faecalibacterium prausnitzii*, and an increase in pro-inflammatory species, including *Escherichia coli*, have been observed. Metabolomic studies reveal decreased short-chain fatty acids (SCFAs) and secondary bile acids, critical for gut homeostasis, alongside elevated pro-inflammatory metabolites. The gut microbiota interacts with host immune pathways, influencing morphogens, glycosylation, and podoplanin (PDPN) expression. The disruption of glycosylation impairs mucosal barriers, while aberrant PDPN activity exacerbates inflammation. Additionally, microbial alterations contribute to oxidative stress, further destabilizing intestinal barriers. These molecular and cellular disruptions underscore the role of the microbiome in IBD pathophysiology. Emerging therapeutic strategies, including probiotics, prebiotics, and dietary interventions, aim to restore microbial balance and mitigate inflammation. Advanced studies on microbiota-targeted therapies reveal their potential to reduce disease severity and improve patient outcomes. Nevertheless, further research is needed to elucidate the bidirectional interactions between the gut microbiome and host immune responses and to translate these insights into clinical applications. This review consolidates current findings on the gut microbiota’s role in IBD, emphasizing its diagnostic and therapeutic implications, and advocates for the continued exploration of microbiome-based interventions to combat this debilitating disease.

## 1. Introduction to Inflammatory Bowel Disease (IBD)

Inflammatory bowel disease (IBD) is a destructive intestinal disease with three main subtypes: Crohn’s disease (CD), ulcerative colitis (UC), and IBD unclassified (IBD-U). They affect millions worldwide [1]. They are heterogeneous and complex disease processes that have differences and overlapping characteristics and risk factors. The causes of IBD are thought to be multifactorial. In terms of environmental factors, many established clinical risk factors exist [2]. Cigarette smoking is correlated with fewer symptoms in UC, but worse in CD [3]. In contrast, antibiotic use at an early age and NSAID are associated with an increased risk of IBD later [4]. Furthermore, diet and microbiota have been linked with IBD. There is also a hereditary link; prior clinical studies have shown a >10% rate of IBD family history in patients with IBD [5]. Genome-wide studies estimate 230 alleles associated with an increased risk of IBD, many of which play a role in host–microbiome interactions [6]. The microbiome plays a key role in IBD environmental, hereditary, molecular, and immunologic risk factors.

Prior studies have shown that the microbiome is distinct between CD, UC, and healthy controls [7]. They have different clustering patterns of common bacteria which have been studied using metagenomic sequencing, with IBD patients often having more pro-inflammatory bacteria [7]. As part of the Integrative Human Microbiome Project, the microbiota of IBD patients was extensively studied and shown to have an overall increase in facultative anaerobes and less obligate aerobes [1]. It is thought that this functional dysbiosis—or imbalance of harmful and helpful bacteria—contributes to abnormal immune function and disrupted mucosal barrier contributing to intestinal inflammation [8]. For instance, when the tight junctions of the intestinal epithelial cell mucosal barrier are damaged, the intestine becomes vulnerable to inflammation [9].

Oxidative stress and increased glycosylation also can lead to the disruption of the intestinal mucosal barrier [10]. Various metabolites are harmful to the intestine and are found to be associated with IBD. In a recent study, stool from IBD patients was found to have increased levels of sphingolipids and bile acids [11]. The excess of primary bile acids is likely related to the decreased recycling of bile acids due to terminal ileal disease as well as a disruption of bile acid metabolism due to the IBD microbiome. In the same study examining IBD patients’ stool, IBD patients were also shown to have overall less diverse microbial metabolites, consistent with the less diverse microbiome [12]. The dysregulated metabolism of bile acids affects the levels of sphingolipids and amino acids in fatty acids. Recent work has shown that deoxycholic acid, a bile acid, can lead to intestinal ecologic imbalances that sequentially cause intestinal inflammation and damage the intestinal mucosa barrier. Furthermore, the essential amino acid tryptophan can be metabolized into indole-metabolites, which have been shown to disrupt intestinal permeability and affect mucosa by binding to a receptor called progesterone X. The microbiome involved in these metabolic processes and thus can indirectly alter the intestinal lining [12].

In contrast, there are beneficial bacteria that assist with gut homeostasis and health, such as those that produce short-chain fatty acids (SCFAs) during fermentation [13]. SCFAs have been shown to help promote B cell development and T regulatory cell expansion. Microbe dysbiosis and an increase in inflammatory intestinal cells can result in a decrease in SCFAs [14]. The production of butyrate, a four-carbon short-chain fatty acid produced by *F. prausnitzii*, is anti-inflammatory and inhibits signal transduction of IL-6 [11]. Additional metabolites like bacteriocins, produced by bacteria like lactobacilli, can inhibit *Listeria* infections [15]. IBD patients have been found to have a decrease in triacylglycerols and tetrapyrroles, which are more protective [12,13]. Prior clinical studies have highlighted the importance of diverse bacterial flora, such as when studying the use of probiotics. A recent retrospective study showed a significant decrease in adverse events—such as the need for surgery, systemic steroids, or hospitalization in IBD patients taking probiotics [16].

As our understanding of the molecular pathogenesis of IBD, including the interplay of the microbiome, continues to deepen, so will the community’s ability to develop effective, targeted medical therapies [17]. There are various targeted medical therapies, such as monoclonal antibodies and biomaterials, under investigation. Various monoclonal antibodies are currently being used to treat patients [18]. Those include anti-TNF antibodies: infliximab (“Remicade”, “Remsima”, “Inflectra”), adalimumab (“Humira”), and golimumab (“Simponi”). Two newer antibodies have been recently introduced, including anti-integrin antibody vedolizumab (“Entyvio”) and ustekinumab (“Stelara”). These agents have revolutionized maintenance care for IBD patients. Studies continue to optimize the dosing and application of these existing agents, as well as continually creating new targeted antibody therapies [18]. In addition, the use of biomaterials has been introduced to allow the controlled release of medications in conditions unique to IBD intestine. These delivery vehicles include hydrogels, nanoparticles, nanofibers, and hybrid systems [19]. The medications that are being delivered are similar to those already used to treat IBD, such as aminosalicylates and corticosteroids, as well as live probiotics [19]. These smart bionanomaterials show great promise in helping optimize the delivery of medications to the inflamed intestine of IBD patients.

## 2. Composition of Gut Microbiota in an Inflammatory Bowel Disease (IBD) Patient and a Healthy Human

A search was performed via PubMed with the parameters “gut microbiota in IBD vs. healthy” and “gut dysbiosis in IBD vs. healthy”, and the resulting articles were assessed for relevance. All age groups (pediatric and adult) were included, as were all disease states. Sources were only included if they assessed both gut microbiota in IBD and healthy controls. Additionally, a systematic review [20] was found, and its sources were assessed as well. A total of forty-one articles were found, which are summarized in Table 1.

Of these articles, sixteen examined patients with CD only [2,3,4,5,6,7,8,9,10,11,12,13,14,15,16,17], two examined patients with UC only [20,21], and the remaining twenty-three examined both patients with CD and patients with UC [22,23,24,25,26,27,28,29,30,31,32,33,34,35,36,37,38,39,40,41,42,43,44]. The majority included adult patients only [2,3,4,5,6,7,8,9,10,11,12,13,20,21,22,23,24,25,26,27,28,29,30,31,32,33,34,35,36,37,38,39,40], though one included a patient as young as 16 years old [14]. Seven included pediatric patients only [15,16,17,41,42]. The method of assessing the gut microbial composition varied between studies, with thirty using fecal/stool samples alone [3,4,7,8,11,12,13,14,15,17,18,19,20,21,22,23,24,25,27,29,30,31,32,34,35,36,37,40,41,42,43,44], five using mucosal biopsy alone [10,16,28,33,38], and three using both fecal/stool sample and mucosal biopsy [5,9,39]. Ghoshal et al. [26] specifically focused on small intestine bacterial overgrowth (SIBO) in CD/UC and used a glucose hydrogen breath test to assess this. Pedamallu et al. [2] targeted ileal deep-tissue microbiome with paraffin blocks of resected ileum.

The majority of the studies assessed the bacterial microbiota [2,3,4,5,6,7,8,9,10,11,12,13,14,15,16,17,21,22,23,24,25,26,27,28,29,30,31,32,33,34,35,36,37,38,43,44], three assessed the fungal microbiota [10,20,37], and one assessed the viral microbiota [42] of IBD patients compared to healthy controls. Overall, the results of the bacterial studies were quite varied, though there were a few findings that were consistent across multiple articles. The most consistent finding was decreased diversity/richness of microbiota in both CD and UC compared to healthy controls [3,4,5,6,8,10,12,17,28,29,34,41,43]. Eun et al. [5] found that this decreased diversity was only present in the fecal samples, and there was a difference between mucosal biopsy samples from healthy controls. Assa et al. [16], who specifically assessed new-onset pediatric CD, did not find a difference in diversity between CD patients and healthy controls. Another consistent finding was the decreased abundance of *Faecalibacterium*, specifically *F. prausnitzii*, in both CD and UC [6,7,9,11,14,15,17,21,22,34,38,39,43], though Assa et al. [16] found increased *Faecalibacterium prausnitzii* species. It is known that a decreased *Firmicutes/Bacteroidetes* ratio is associated with IBD [45], which is further supported by the findings of decreased *F. prausnitzii*. Both conflicting findings from Assa et al. [16] may suggest that the microbiota in new-onset pediatric CD patients differ from patients with the longer-standing disease. Further findings include increased *Enterococcus* sp. in CD and IBD [13,15,17,23,24,36,40,46,47,48,49,50,51,52] and increased *Escherichia coli* in CD and UC [9,13,22,28,30,33,36,43,53,54,55,56,57].

Studies evaluating fungal and viral composition were few, limiting the conclusions that can be drawn from these sources. Of the three studies that assessed the fungal microbiota [10,20,37], all found that the overall fungal level was increased, but *Saccharomyces cerevisiae*, specifically, was decreased. Liang et al. [42], in assessing the viral microbiome of very early onset IBD, found no significant difference in the overall viral load between IBD patients and healthy controls, but IBD patients had an increased ratio of *Caudovirales* to *Microviridae* compared to healthy controls and increased *Anelloviridae* prevalence. Other findings of note include the following: samples of deeper intestinal tissue did not show increased *Enterobacteriaceae*, *Pasteurellaceae*, *Veillonellaceae*, and *Fusobacteriaceae* families, which had previously been seen in upper mucosal layers [2,58,59]; increased bacterial variability in IBD vs. healthy control fecal/stool samples but decreased variability in mucosal samples [5]; mucosal microbiota of CD patients had a lower proportion of core species of bacteria and had a higher proportion of rare species [6]; and a decrease in bacterial diversity in IBD patients with ileal disease but no decreased diversity in IBD patients with the colonic disease [31]. The remaining results are summarized in Table 2.

## 3. How Gut Microbiota Affect Morphogen in IBD

Morphogens are molecules that spread from localized sources to form concentration gradients and control cell fate in a concentration-dependent manner [60,61,62]. Morphogens are signaling molecules that regulate the pattern of tissue development in the process of morphogenesis [63]. They form gradients and elicit different cellular responses based on their concentration, thus providing positional information to cells [64,65]. There is a growing body of research examining the association between morphogens, gut microbiota, and IBD. Morphogens, which are signaling molecules that regulate tissue development and cellular differentiation, have been implicated in the pathogenesis of IBD through their influence on intestinal epithelial cells and the immune response. One study highlights the role of various morphogens, such as members of the Wnt/β-catenin signaling in maintaining intestinal homeostasis and their dysregulation in IBD patients [66]. Disruption in these signaling pathways can lead to altered cell proliferation, differentiation, and apoptosis, contributing to chronic inflammation, which is a hallmark characteristic of IBD [67,68].

Furthermore, recent research suggests that morphogens like Sonic Hedgehog (Shh) and Bone Morphogenetic Proteins (BMPs) can influence the gut microbiota composition, which, in turn, affects the inflammatory state of the gut. This interaction between morphogens and gut microbiota underscores the complexity of IBD pathogenesis and highlights potential therapeutic targets [69,70]. Abnormal signal transduction between the epithelial cells and nearby immune cells is thought to exacerbate this immune dysregulation, potentially contributing to the chronic inflammation characteristic of IBD [71]. In patients with IBD, significant microbial alterations occur, contributing to disruptions in the mucosal barrier. These changes compromise the intestinal lining’s integrity, leading to increased permeability and susceptibility to inflammation [72]. The tripartite communication network between immune cells, intestinal epithelial cells, and stromal cells is crucial in maintaining gut homeostasis and responding to inflammatory stimuli in diseases like IBD [73]. In IBD, chronic inflammation perpetuates a complex interplay between the immune system, epithelium, and endothelium, driving tissue damage and fibrosis. Understanding the intricate molecular mechanisms behind these processes is crucial for developing therapeutic strategies that effectively target inflammation and mitigate fibrotic complications in IBD patients [74]. IBD may arise due to deficiencies in the intestinal mucosa’s protective mechanisms and its ability to appropriately repair after injury. Various cell populations coordinate these functions through a diverse range of growth factors, which regulate cell proliferation, immune responses, and tissue remodeling [75]. Microbes can influence intestinal physiology by inducing cell turnover and altering overall organism function, thereby affecting intestinal stem cell (ISC) activity, which is coordinated through various signaling pathways. *Lactobacillus reuteri* promotes intestinal cell growth, repairs epithelial damage, reduces inflammation, and maintains intestinal health by regulating the Wnt/β-catenin pathway [76]. *Ruminococcacea* can produce short-chain amino acids that promote small intestinal and villus growth by activating the Wnt/β-catenin pathway to stimulate the proliferation of intestinal stem cells [77].

## 4. How Gut Microbiota Affect Podoplanin in IBD

Podoplanin (PDPN) is a glycoprotein expressed in various tissues, including lymphatic endothelial cells and epithelial cells of the intestine [78]. It plays a crucial role in lymphangiogenesis and immune responses. In the context of IBD, PDPN has emerged as a significant player in the pathophysiology of the disease. PDPN expression is upregulated in inflamed tissues, where it contributes to the formation of lymphatic vessels and facilitates the transport of immune cells [79]. This process is essential for maintaining immune homeostasis and tissue repair during inflammation. Emerging evidence suggests that gut microbiota may influence PDPN expression. In summary, dysbiosis can lead to increased PDPN levels, which in turn may exacerbate inflammation and tissue remodeling in IBD [80,81]. The intricate interplay between gut microbiota, immune responses, and PDPN expression underscores the complexity of IBD pathogenesis. Dysbiosis influences the expression of key molecules like PDPN, which are involved in immune regulation and tissue repair. Dysbiosis also contributes to chronic inflammation in the intestinal tract. Since PDPN expression is known to be upregulated in inflamed tissues, the altered microbiota can indirectly affect PDPN levels by promoting inflammation [82,83].

**Figure 1:**
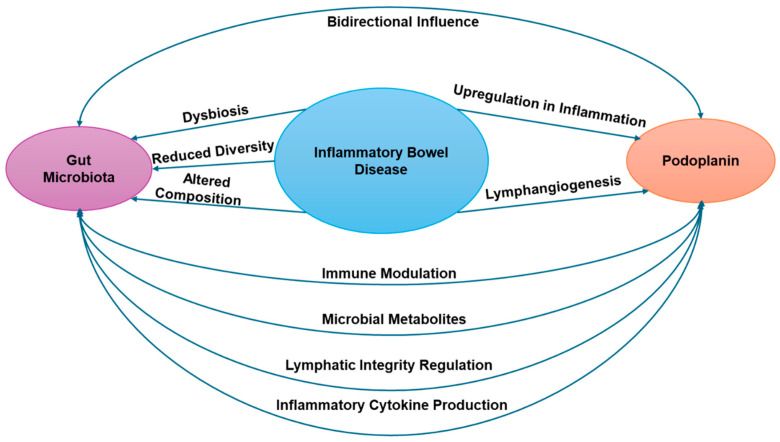
It illustrates the effect of gut microbiota on podoplanin (PDPN) in IBD.

In IBD, there is impaired lymphatic clearance function, which causes an obstructed flow of interstitial fluid and immune cells [78]. Gut microbiota is an important regulator of lymphatic integrity [78]. In investigating the impact of IBD on UC, researchers evaluated the structural and function changes in the intestinal lymphatic vessels. Lack of lymphatic draining increased the severity and inflammation of UC due to reduced survival rate, increased injury, and immune cell infiltration [84]. However, when lymphatic drainage improves, consequently, the gut microbiota diversity significantly decreased [85]. To be more specific, the colitis model has been shown to cause significant gut microbiota changes in reduced abundance of Firmicutes and increased Bacterioidate at phylum levels in fecal samples, which most likely means high PDPN expression. The microbiota changes together with the restored balance between immune cells and associated cytokines, contributing to resolving colonic inflammation, which most likely means low PDPN expression in stromal cells and lymphatics. Impaired lymphatic clearance in IBD exacerbates inflammation and immune cell infiltration. Interestingly, when lymphatic drainage improves, gut microbiota diversity decreases significantly [86]. This suggests a bidirectional relationship between lymphatic function (which involves PDPN) and gut microbiota composition. Gut microbiota has been identified as an important regulator of lymphatic integrity. Given that PDPN is crucial for lymphatic vessel formation and function, changes in the microbiota likely influence PDPN expression and activity in lymphatic endothelial cells. Figure 1 of this paper illustrates the effect of gut microbiota on podoplanin in IBD.

The gut microbiota shapes the immune response in the intestine [86]. As PDPN is involved in immune cell trafficking, alterations in the microbiota could affect PDPN expression or function through immune-mediated mechanisms. Improved lymphatic drainage, which is associated with PDPN function, leads to changes in gut microbiota composition. This shift, along with the restored balance of immune cells and cytokines, contributes to resolving colonic inflammation. This resolution likely results in decreased PDPN expression in stromal cells and lymphatics, as PDPN is typically upregulated during inflammation [86].

## 5. How Gut Microbiota Affect Glycosylation in IBD

Glycosylation involves the combination of monosaccharides that are attached to other macromolecules forming glycoproteins or glycolipids. These play an integral role in cell adhesion, growth, death, and migration as well as embryonic development, homeostasis, and immunity [10]. Type O-mucin is the major glycan in the gut, making up 80% of the most abundant intestinal mucin: MUC2 [10]. This mucus layer is especially important because it provides a physical barrier against pathogens and toxic substances, relays signals that regulate immune system function, serves as a source of nutrients for commensal organisms, and forms the bridge between luminal contents and the epithelial barrier [87]. Intestinal mucus, under normal conditions, can protect the underlying epithelium via different forms of glycosylation, one of which is O-linked glycosylation [88]. The O glycans that make up MUC2 studied in colitis mouse models had three primary deficiencies: terminal sialylation, fucosylation, and sulfation [88].

Sialylation, the addition of a sialic acid residue, increases the flexibility and viscosity of the polymers and helps prevent direct contact between harmful pathogens and substances within the lumen and the epithelial layer [89]. Sialyltransferase ST6GALNAC1 (ST6) in goblet cells protects against bacterial proteolysis of mucins by catalyzing the terminal sialylation of glycans [87]. Mouse models with knock-in ST6 deficiency had more severe colitis [87]. Additionally, three patients with early onset IBD were found to have biallelic germline loss of the ST6 gene [87]. Sialic acid residues attached to cell-surface glycoproteins also serve as ligands for immune receptors, regulating their activation and response [90]. Thus, altered patterns of glycosylation, specifically sialylation, result in the dysregulation of immune tolerance and can be part of the pathophysiology of autoimmune diseases such as IBD [87,91]. Fucosylation has also been shown to be altered in states of intestinal inflammation [92]. The process of adding fucose is responsible for the formation of ABO blood-type antigens, called the H antigen [92]. In the intestinal epithelium, the enzyme FUT2 is responsible for this antigen serving as a binding site for certain commensal organisms, as well as a source of energy for others [92]. The increased fucosylation of the intestinal epithelium has been shown to decrease gut colonization by opportunistic pathogens such as *E. faecalis* [93]. Mouse models have confirmed that commensal bacteria interactions with innate lymphoid cells upregulate fucosylation that resists microbial dysbiosis [92,93]. Fucosylation additionally has direct anti-inflammatory properties by downregulating macrophage M1 polarization and inhibiting NLRP3 inflammasome and NF-kB activation [92,94]. Therefore, alterations in the fucosylation of the mucus layer and intestinal epithelial cells are both pro-inflammatory and detrimental to intestinal homeostasis. Sulfation is another form of glycosylation that has anti-inflammatory effects. Sulfation can be induced by the gut microbiome and was shown to be decreased in IBD [95,96,97]. Like sialylation and fucosylation, sulfation is another modifier of intestinal mucins, and mouse models with decreased sulfation were found to have increased intestinal permeability, more severe DSS-induced colitis, and increased leukocyte trafficking to the gut [92]. Population-level studies of IBD have also found that North American and European patients have reduced mucus sulfation compared to South Asians and have a higher incidence of IBD [92].

Inflammatory conditions such as CD or UC create an environment within the intestinal lumen that preferentially allows certain parts of the gut microbiota to flourish at the expense of others [98]. It is hypothesized that this microbial dysbiosis can affect the glycosylation pathways in the gut [98]. There is a complex relationship between the microbiome, glycosylation pathways, and inflammatory states, but in studies of both human and mouse populations with colitis, a few bacterial species were consistently more abundant in patients with IBD, including *Escherichia coli*, *Ruminococcus gnavus*, *Bacteroides fragilis*, and *Clostridium* spp., as well as *Clostridium innocuum*. These were increased even with variations in genetics and diet among the different cohorts of patients [99]. Another study using mouse models found that mice with colitis induced by dextran sulfate sodium (DSS) had a significantly increased prevalence of *Ruminococcus* spp., *Bacteroides* spp., *Salmonella* spp., and *Escherichia* spp. [100,101,102]. Other species identified to be increased in IBD were *Shigella* spp., *Fusobacterium nucleatum*, and *Akkermansia muciniphila* [103]. Some studies have even shown higher incidences of *Clostridium difficile*, *Mycobacterium avium*, and *Listeria monocytogenes* as part of the disturbances in gut microbiota homeostasis [103,104]. The presence of all of these types of pathogenic bacteria triggers inflammation in the colon that leads to decreased activity of goblet cells by inhibiting the expression of KLF4 and TFF3 [105].

Some of these strains possess the capacity to synthesize sialidases (*Ruminococcus gnavus*, *Bacteroides*, *Clostridia*, *Streptococci*, and so on), which are enzymes responsible for hydrolyzing sialic acid residues from glycoproteins and glycolipids [89]. The reversal of the effects of sialylation destabilizes the mucus barrier and further promotes the growth of pathogenic species. For example, *Bacteroides vulgatus* expresses sialidase, which provides *Escherichia coli* with sialic acid residues [100]. The subsequent proliferation of this species results in chronic inflammation of the intestinal lining through the activation of dendritic cells [100]. Some prominent examples of medications that are sialidase inhibitors are the anti-influenza medications Oseltamivir and Zanamivir. The target of these medications, neuraminidase, contains sialidase activity that facilitates the budding of progeny virions [101]. Potential antibiotic targets have been studied in *Clostridium perfringens*, which have sialidase activity in the form of NanH, NanI, and NanJ [102]. These enzymes cleave sialic acid residues as a carbon source while also promoting host cell adherence and endothelial barrier dysfunction [102]. Nani in particular increases the potency of the alpha toxin which leads to gas gangrene and other toxins that promote intestinal disease [102]. Two known targets of these sialidases are Siastatin B and N-acetyl-2-3-dehydro-2-deoxyneuraminic acid, both of which have been shown in studies to diminish the pathogenicity of *C. perfringens* [102]. Other strains possess various forms of sulfatases which reverse the process of sulfation, leading to similar impairments on the intestinal barrier [104].

On the other end of the spectrum are findings of commensal species that were decreased, including *Faecalibacterium prausnitzii*, *Roseburia intestinalis*, *Eubacterium hallii*, *Gemmiger formicilis*, *Eubacterium rectale*, and *Ruminococcus bromii* [99]. These are all known for the production of butyric acid, a metabolite with known anti-inflammatory properties [99,106]. Additional commensal species involved in mineral metabolism (*Collinsella aerofaciens*), bile acid metabolism (*Ruminococcus torques*), and urea cycle metabolism (Bifidobacterium longum) were also significantly decreased [99]. Anti-inflammatory bacteria such as *Alistipes putredinis*, *Asaccharobacter celatus*, and *Gemmiger formicilis* were also found to be decreased. The decline of *A. putredinis* was correlated with a corresponding increase in the number of *Candida albicans*, resulting in increased Th17 cell differentiation and further inflammatory changes [99]. *Asaccharobacter celatus* in particular had been identified in previous studies to decrease inflammation in conditions such as autoimmune encephalitis [99].

Some of these commensal bacteria also compete with pathogens for sialic acid utilization or produce metabolites that inhibit pathogen adhesion, thereby contributing to host defense against infections [89,107]. Furthermore, some commensal bacterium expresses sialidase as well, such as *Bacteroides fragilis*, which expresses NanH [89,108]. NanH has been shown to assist with the recovery of commensal bacteria after the administration of antibiotics [89,108]. Commensal bacteria have also been shown to promote anti-inflammatory states by upregulating Treg cells [109].

## 6. How Gut Microbiota Affect Bile Acid Levels in IBD

Bile acids (BAs) are hydroxylated, amphipathic steroid acids synthesized in the liver’s peroxisomes from cholesterol [110]. They play a crucial role in the digestion and absorption of dietary fats and fat-soluble vitamins [111]. BAs are conjugated to hydrophilic amino acids glycine or taurine, resulting in primary or conjugated BAs, such as cholic acid (CA) and chenodeoxycholic acid (CDCA), and their tauro- and glycoconjugated versions [111]. Humans predominantly use glycine for conjugation, while rodents primarily use taurine [110]. Primary BAs are secreted into the bile and stored in the gallbladder [112]. Upon food intake, cholecystokinin triggers gallbladder contraction, releasing primary BAs into the duodenum [113]. There, they act as surfactants, emulsifying fats into micelles to facilitate the digestion and absorption of dietary lipids, cholesterol, and fat-soluble vitamins [111]. More than 95% of primary BAs are reabsorbed from the terminal ileum and transported back to the liver via enterohepatic circulation [114]. In the liver, they inhibit cholesterol and further BA biosynthesis [114]. However, some primary BAs reach the colon, where gut bacteria transform them into secondary BAs, such as deoxycholic acid (DCA), ursodeoxycholic acid (UDCA), and lithocholic acid (LCA) [115]. These secondary BAs have strong antimicrobial properties and help regulate gut bacterial communities and host physiology [116]. BAs activate nuclear and plasma membrane receptors, including farnesoid X receptor (FXR) and G protein-coupled receptor (TGR5), which control BA synthesis and metabolism [117]. These receptors also help regulate glucose homeostasis, lipid metabolism, and energy expenditure [118]. Additionally, BAs influence immune responses by engaging these receptors, which are expressed on immune cells like macrophages, dendritic cells, and natural killer T (NKT) cells [118]. Both primary and secondary BAs enhance the antimicrobial properties of immunoglobulin A (IgA), inhibiting bacterial growth and protecting against infections in the biliary tract [114]. The regulation of the bile acid (BA) pool exemplifies how microbial metabolism interferes with the host [116]. The processes of deconjugation, oxidation/epimerization, (7-α-) dehydroxylation, and esterification of BAs by the intestinal microbiota can significantly alter their physicochemical properties [116]. These changes can impact the microbial toxicity of BAs and their absorption in the intestine [116]. In a preclinical study, bile acid homeostasis was assessed in a four mice model, and the control group was treated with antibiotics and compared with untreated mice [119]. The results showed that the attenuation of gut microbiota by the antibiotics increased absorption and decreased synthesis of bile acids [119], [120]. Patients with IBD show reduced microbial diversity and an abnormal microbial composition, characterized by a decrease in Firmicutes (including bile acid-metabolizing bacteria) and an increase in Proteobacteria [121]. This dysbiosis hampers bile acid transformation, resulting in higher levels of primary and conjugated bile acids and lower levels of secondary bile acids [121]. Both gut dysbiosis and altered bile acid profiles impair gut barrier function and immunity [121]. Table 3 of this paper represents various findings based on pre-clinical and clinical studies.

In a preclinical experimental model, the decreased expression of the apical sodium-dependent bile acid transporter (ASBT) was seen [122]. Similarly, human IBD studies concluded that the inflamed ileum can disrupt the enterohepatic recirculation of bile acid, possibly by repressing the ASBT promoter [122]. Another preclinical experiment, in which ASBT regulation was studied in IL-1beta- treated IEC-6 and Caco cells and indomethacin-treated rats, showed that it led to direct reductions in ileal ASBT messenger RNA and protein levels [128]. In IBD patients, the decreased microbial abundance in the distal ileum and colon results in elevated conjugated bile acids accumulation and decreased secondary bile acids [128]. Similar results have been observed in many colitis rodent models. In the trinitrobenzene sulfonic acid (TNBS)-induced colitis model, reduced expression of bile acid (BA) transporters led to an increase in BAs accumulating in feces, thereby suppressing BA recycling [123]. In another preclinical study, rats with dextran sodium sulfate (DSS)-induced colitis also exhibited increased fecal cholic acid (CA) [124]. Notably, pediatric patients with IBD had a significantly reduced potential for BA production in their microbiome [125]. Additionally, colectomy-treated patients with UC showed decreased levels of deoxycholic acid (DCA) and lithocholic acid (LCA), along with fewer genes needed for converting primary to secondary BAs in their pouches [126]. In the context of colitis-associated cancer (CAC), a study found reduced fecal BAs and decreased transformation of primary to secondary BAs, along with a downregulated gut–liver farnesoid X receptor–fibroblast growth factor 15 (FXR-FGF15) axis in the CAC mouse model [127].

## 7. How Gut Microbiota Affect Immunity in IBD

In IBD, several factors affect the immune system [8]. The dysfunction of the gut microbiota is noted throughout both subtypes of IBD. The microbiome is the community of microorganisms that live on and inside us. These microorganisms include bacteria, fungi, viruses, and archaea [8,10]. The human microbiome is vast. It plays an important role in our health, influencing everything from digestion and immunity to mood and weight. The microbiome is constantly changing and is influenced by a variety of factors, including diet, exercise, and medications. A healthy microbiome is essential for good health, and imbalances have been linked to several diseases, including obesity, diabetes, and IBD. In this review, we will look at how the gut microbiota affects immunity in IBD through lab and clinical studies using animal and human subjects.

Dysbiosis in gut microbiota is linked to IBD, with decreased beneficial bacteria and increased pathogenic microbes [8]. Experiments involved mice to study the effects of Polysaccharide A (PSA) from Bacteroides *fragilis* on CD4^+^ T cells and intestinal inflammation. Specific pathogen-free and germ-free IL10(−/−) mice were used to assess the impact of *Akkermansia muciniphila* on intestinal inflammation [8]. PSA directs CD4^+^ T cell development and induces anti-inflammatory Tregs, protecting animals from experimental colitis through IL-10-producing T cells [8]. A *muciniphila* helps improve the gut barrier through its outer membrane protein Amuc_1100 [8] A study involving 46 IBD patients and 20 control patients to analyze the abundance of *Akkermansia muciniphila* showed significantly reduced levels in IBD patients [8]. 

Altered glycosylation patterns in the gut mucosa are associated with IBD, leading to changes in bacterial colonization and inflammation [10]. Mice studies show that intestinal glycans regulate bacterial distribution in the gut. Disruption in glycan patterns leads to bacterial overgrowth in specific areas, contributing to IBD [10]. Mice lacking specific glycosyltransferases (e.g., FUT2 non-secretor mice) exhibit changes in gut microbiota composition and increased susceptibility to inflammation, mimicking human IBD conditions [10]. MUC2 mucin glycosylation is crucial for maintaining gut barrier function and immune tolerance [10]. Mice with defective MUC2 glycosylation develop spontaneous colitis, providing a model for studying colitis-associated cancer [10]. IL-22 induces glycosyltransferase transcription in epithelial cells, affecting gut microbiota and inflammation levels. Table 4 of this table represents the summary profile of various pre-clinical and clinical studies of how gut microbiota affects immunity in IBD.

Human studies examined the gut mucosa of patients with active UC and identified altered O-glycosylation profiles. These alterations are associated with increased inflammation and are reversible [10]. IBD patients with different FUT2 genotypes show that those with FUT2 non-secretor status have a distinct gut microbiota composition, linking genetic glycosylation patterns to microbial diversity and IBD susceptibility [10].

Neutrophils play crucial roles in the immune response and inflammation in IBD [129]. They can contribute to both the exacerbation and resolution of inflammation through various mechanisms [129]. They interact with the gut microbiota, influencing bacterial composition and activity [129]. To identify and validate biomarkers for monitoring disease activity in IBD patients [129], researchers measured levels of fecal calprotectin and myeloperoxidase in IBD patients and correlated these with disease severity and endoscopic findings [129]. Elevated levels of these biomarkers were found to be associated with increased neutrophil activity and higher disease severity, suggesting their utility in monitoring IBD [129].

IBD patients, including those with UC and CD, exhibit reduced diversity and abundance of beneficial bacteria and an increase in pathogenic species [130]. In human studies, 41 IBD patients (18 with UC) and 23 with CD) and 20 healthy controls were recruited [130]. IBD patients were divided into active (IBD-A) and remissive (IBD-R) groups based on disease activity, assessed using the Mayo ulcerative colitis endoscopic index for UC and the Crohn’s Disease Activity Index for CD [130]. Fecal samples were collected, stored, and processed for DNA extraction [130]. The Chao index, ACE index, Shannon index, and Simpson index were used to assess the diversity and abundance of gut microbiota [130]. Both UC and CD patients showed significantly lower diversity and abundance of gut microbiota compared to healthy controls [130].

## 8. How Gut Microbiota Affect Various Pathways, Receptors, and Gene Signaling and Mechanisms in IBD

A healthy gut mucus layer is necessary to maintain a balanced relationship between a host and gut bacteria [131]. This layer acts as a protective barrier and allows for beneficial interactions between the microbiome and intestinal lining [132]. Problems with the mucosal layer are associated with an increased risk of developing IBD [131].

NOD2 is a gene that is linked to IBD, acting as a sensor in cells and being able to recognize a part of bacteria called muramyl dipeptide, also known as MDP [133]. By detecting the MDP portion, NOD2 activates NF-kB. It works with other inflammasomes to release IL-1B, a pro-inflammatory cytokine [133]. In people with IBD, there is often dysbiosis, characterized by fewer bacteria that produce SCFAs and lower levels of butyrate (BT). These changes are also associated with an increase in pro-inflammatory immune cells in the gut lining [133]. When kids with CD lose bacteria that make butyrate, it reduces butyrate production. This can hurt mitochondria, leading to the formation of ROS [134].

A study conducted on people with CD showed that NOD2 mutations can weaken the activation of NFkB, and, in return, the lack of bacterial response caused excess inflammation [135]. Interestingly, other inflammatory genes like NLRP3 and interferons were sometimes more active instead [136]. Another study on mice lacking a functional NOD2 gene showed trouble producing the cytokines when exposed to MDP, leading to persistent inflammation through an alternative pathway [135]. IBD is thought to be linked to cellular stress pathways as well. Studies identified genetic variations in autophagy genes (IRGM and ATG16L1), which are genes that break down cellular waste and help with cell maintenance and metabolism [137]. For example, changes in the ATG16L1 gene are linked to a higher risk of CD [137]. One study was carried out where the CD-associated ATG16L1 variant illustrated mice with defects that are likely associated with an increased risk of IBD in humans, such that the immune cells of these mice would produce excess IL-1B when inflammasomes were activated [135]. In cells with less ATG16L1, more cytoplasmic vesicles were seen in Paneth cells under a microscope, like what is observed in CD patients [136]. B-cells in IBD patients cause gut inflammation by making many types of antibodies like anti-pancreatic antibodies, perinuclear antineutrophil cytoplasmic autoantibodies (pANCA), anti-Saccharomyces cerevisiae mannan antibodies (ASCA), which can ultimately cross-react with intestinal bacteria [138].

In people with IBD, their gut bacteria show reduced levels of Firmicutes and Bacteroidetes, while Proteobacteria and Actinobacteria are more abundant [104]. Specifically, there is less production of SCFAs and bile acid breakdown, and higher levels of redox potential and hydrogen sulfide (H_2_S) production [139]. These metabolic changes are linked to problems in human cell pathways, like difficulties in detoxifying H_2_S, transporting and using SCFAs, and dealing with high redox potential in the gut [104]. UC is characterized by a strong Th2 immune response, leading to increased production of cytokines like IL-5 and IL-13, which contribute to inflammation and tissue damage [140]. The balance between Treg cells (which suppress inflammation) and Th17 cells (which promote it) is crucial in UC [140]. Patients often show a decrease in Treg cells and an increase in Th17 cells, which correlates with disease severity [139]. This imbalance, influenced by factors like TGF-β and specific cytokines, has become a target for potential UC treatments [140].

## 9. How Gut Microbiota Contribute to Oxidative Stress in IBD

The development of inflammatory diseases like IBD is linked to significant alterations in the gut microbiome [141]. IBD is defined by changes in the intestinal microbiota and an inappropriate immune response to environmental factors [142]. These chronic IBDs stem from a complex disturbance of immunological homeostasis [143]. In a healthy organism, homeostasis maintains a balance of pro- and anti-inflammatory mechanisms, with temporary inflammatory reactions occurring only in response to actual infections [144]. In the gastrointestinal tract, bacteria directly interact with the host, striking a delicate balance between preventing unnecessary inflammatory reactions and ensuring the immune system is activated when pathogens invade [143]. Bacterial-derived metabolites can disrupt the stable redox balance maintained by a healthy microbiome, leading to an imbalanced redox environment that affects the immune system by altering intracellular signaling and promoting inflammation [145].

Reactive oxygen species (ROS) are enzymatic byproducts that act as intracellular and intercellular messengers, modifying proteins such as p53, Jun, Fos, and NF-κB subunits to either stimulate or inhibit oxidation [143]. When pathogenic microbiota is introduced, however, the redox balance tips into the pro-oxidative state, inducing inflammation [144]. Cellular ROS production starts with an electron transfer to oxygen, forming highly reactive and short-lived radical oxygen (O_2_^−^) molecules [143]. Their charge restricts them from crossing cellular membranes, causing localized oxidative damage [146]. Meanwhile, mammalian cells produce nitric oxide (NO) via the oxidation of one of the terminal guanidino nitrogen atoms of L-arginine, a reaction catalyzed by the enzyme NO synthase (NOS) [147]. Though less reactive than ROS, NO accumulation leads to rapid reactions with oxygen radicals to form peroxynitrite (ONOO^−^), which then reacts with substrates causing cellular damage [148].

The immune system’s constant direct contact with the microbiota highlights the crucial role immune cells play in maintaining equilibrium and preventing unnecessary inflammation [143]. However, redox imbalance in colonic tissues has been identified as a significant factor linking these reactive molecules to the development and progression of IBD, driven by the excessive ROS produced [149]. Intestinal epithelial and immune cells use pattern recognition receptors (PRRs) like Toll-like receptors (TLRs) and nucleotide-binding oligomerization domain-containing protein (NOD) to detect bacteria and their metabolites, facilitating continuous communication between the microbiota and the host [150]. Host cells recognizing bacteria cause the release of O_2_˙^−^ by NADPH oxidases and dual oxidase 2 (DUOX2) [143]. O_2_˙^−^ quickly converted to H_2_O_2_, which then re-enters the intestinal epithelial cells (IEC), altering signal transduction and initiating inflammatory processes [150]. Inflammatory cytokine expression is triggered by altered cellular signaling, causing an increased NADPH oxidase-dependent ROS production and inducing NOS [143]. The resulting peroxynitrite formation destroys the bacteria but also oxidizes host cell membranes, releasing damage-associated molecular patterns (DAMPs) that intensify the inflammatory response [151].

Dendritic cells are antigen-presenting cells that are activated by toll-like receptors (TLRs), and upon activation, the cells switch to glycolysis where the intermediates are shunted into the pentose phosphate pathway, and NO is produced [143]. NO can then react with O_2_˙^−^ to form highly reactive ONOO^−^ which blocks the ETC, leading to increased ROS generation, which can then impact the activation of CD8^+^ and CD4^+^ T cells [152]. Upon T cell receptor (TCR) stimulation, two signals are triggered: a calcium influx into the cytosol and the initiation of an oxidative signal [143]. Calcium influx activates NF-AT, calcium-dependent transcription factors, and triggers the activation of neuronal nitric oxide synthase (nNOS) and epithelial nitric oxide synthase (eNOS) [153]. This leads to a controlled release of O_2_˙^−^, which superoxide dismutase (SOD) then converts to H_2_O_2_, which then activates transcription factors NF-κB and AP-1 [154]. These transcription factors work with NF-AT for the induction of CD95 death ligand and other cytokines, which then control the induction and termination of T-cell immune response [155]. In the B-cell response, the stimulation of B-cells initially triggers ROS production by phagocytic NADPH oxidase, with the signal being extended by mitochondrial ROS production [143]. Inflammatory M1 macrophages are both induced by and produce ROS, while anti-inflammatory M2 macrophages function independently of ROS; however, prolonged high ROS levels and oxidative stress can induce senescence and trigger macrophage cell death [156]. Other immune cells, like leukocytes and monocytes, are subsequently activated, further increasing ROS accumulation and leading to a chain reaction that ultimately increases epithelial permeability [157]. Increased permeability allows bacteria to penetrate the lamina propria, sustaining immune activation and ROS release, which further enhances the pro-inflammatory environment and perpetuates chronic inflammation [143].

**Figure 2:**
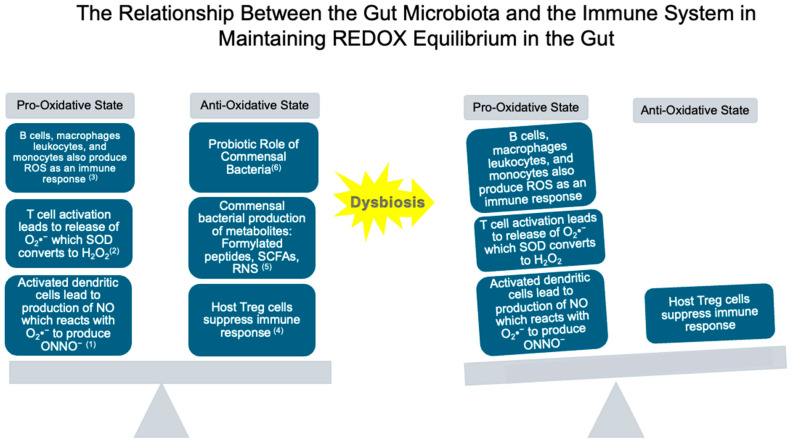
It depicts how REDOX equilibrium is maintained by components of the host’s immune system and commensal bacteria [152]. ONNO^−^ reacts with substrates to cause cellular damage, and it also blocks the ETC, leading to increased ROS generation, which then goes on to influence immune cell activation [150,154]. H_2_O_2_ can re-enter intestinal epithelial cells and initiate inflammation, and it can also activate transcription factors that function to induce and terminate T cell immune response [143,156,157]. B cells, M1 macrophages, leukocytes, and monocytes all function to increase ROS production in response to immune activation [139]. Treg cells function to regulate the immune response, preventing the immune system from overreacting [164]. These metabolites function in an antioxidative capacity and reduce mitochondrial damage and oxidative stress [161]. These bacteria produce their enzymes (SOD and catalase) and metabolites to reduce oxidized molecules [154].

To maintain redox homeostasis in the gut, commensal bacterial communities play a crucial role [158]. These communities comprise an assortment of microbial species including but not limited to *Bacteroides*, *Eubacterium*, *Peptococcaceae*, *Bifidobacterium*, *Escherichia coli*, *Streptococci*, *Staphylococci*, *Lactobacillus*, and *Clostridium perfringens* [159]. Many commensal bacteria also play a part in redox homeostasis via their role as probiotics [160]. They produce their antioxidants, such as SOD and catalase, along with generating antioxidative metabolites that reduce oxidized molecules [161]. Commensal bacteria produce a range of metabolites, including formyl-peptides, reactive nitrogen species (RNS), and SCFAs, that can affect the redox status in the intestine positively [143]. Formylated peptides derived from commensal bacteria bind to G protein receptors on immune and epithelial cells, triggering inflammation and enhancing ROS generation in the gut via NADPH oxidase activation [162]. SCFAs activate the Keap1-Nrf2 pathway, boosting the cellular antioxidant defense system [163]. By enhancing these defenses, SCFAs reduce ROS-induced mitochondrial damage, improve mitochondrial function, and provide protection against oxidative and mitochondrial stress [143]. Commensal bacteria also regulate homeostasis by balancing pro- and anti-inflammatory cytokine production from Th17 and Treg cells [139]. As pro-inflammatory cytokines contribute to increased ROS during inflammation, restoring the balance between Th17 and Treg cells will help prevent excess ROS production in the epithelium [164]. Therefore, targeting gut microbiota to reduce oxidative stress and inflammation in the gut could be a promising therapeutic approach for IBD [165]. These details are summarized in Figure 2 of this paper.

One of the conventional methods of treating IBD is through the use of prebiotics and probiotics to restore gut microbiota diversity [166]. Polyphenols act similarly to prebiotics, increasing the population of beneficial bacteria and boosting their antioxidant potential [167]. An example is the water extract from silver fir (*Abies alba*) wood, which is rich in lignans and phenols and offers therapeutic benefits for various pathological conditions. A study examining the interaction between this extract and ten different *Lactobacillus* species found that it functions as a prebiotic, supporting the growth of several beneficial gut microbiota species [168]. A study investigating the protective effects of rice protein peptides on dextran sulfate sodium-induced colitis in mice showed that polyphenolic compounds enhanced antioxidant signaling pathways, such as Nrf2, which strengthened the intestinal mucosal barrier and supported gut microbiota homeostasis [169]. Another polyphenol is curcumin, which functions to maintain the intestinal mucosal barrier integrity by mitigating the endoplasmic reticulum stress-mediated IEC apoptosis [170]. A recent study confirmed that curcumin mitigates H_2_O_2_-induced oxidative damage by activating the heme oxygenase-1 (*HO-1*) signaling pathway, and they can also influence inflammation by modulating immune cell status [171]. A common adjuvant used to treat IBD is Quercetin, known for its anti-inflammatory and antioxidant effects [166]. Quercetin strengthens the intestinal mucosal barrier, promotes the proliferation of intestinal cells, and promotes the synthesis of GSH and Nrf2 to eliminate oxidative stress [172]. Last is Resveratrol (3,5,4′-trihydroxy-trans-stilbene), a nonflavonoid polyphenol that functions as a powerful antioxidant with antibacterial, anti-obesity, anti-inflammatory, and anticancer properties [173]. Resveratrol and its microbial metabolites can reduce ROS levels, activate Nrf2 signaling, and alleviate oxidative stress, thereby protecting the epithelial barrier and suppressing NF-κB activation and intestinal inflammation [165].

## 10. Conclusions and Future Perspectives

IBD is a destructive intestinal disease with CD, UC, and IBD-U as its three main subtypes. They are heterogeneous and complex disease processes with different and overlapping characteristics and risk factors. Although causes are thought to be multifactorial, it is established that the microbiome plays a key role in IBD environments. Microbes can influence intestinal physiology by inducing cell turnover and altering overall organism function, thereby affecting ISC activity, which is coordinated through signaling pathways involving enterocytes, enteroblasts, enteroendocrine cells, and visceral muscle cells, which can contribute to the pathogenesis of IBD. In IBD patients, gut bacteria show reduced levels of Firmicutes and Bacteroidetes, while Proteobacteria and Actinobacteria are more abundant. Specifically, there is less production of SCFAs and bile acid breakdown, and higher levels of redox potential and hydrogen sulfide (H_2_S) production. These metabolic changes are linked to problems in human cell pathways, like difficulties in detoxifying (H_2_S), transporting and using SCFAs, and dealing with high redox potential in the gut. Hence, there is a huge necessity for studies as well as the development of interventions. Focusing on combating abnormal metabolic alterations as well as their outcomes.

The gut microbiota plays a crucial role in the pathogenesis and management of IBD. Therapeutic strategies aimed at restoring microbial balance, such as probiotics, prebiotics, and dietary interventions, hold promise for improving patient outcomes. Further research is needed to continue understanding how the microbiome continues to interfere with gut health to better target interventions. There is a delicate balance between the intestinal barrier, the gut microbiome, and the biochemical processes such as glycosylation to promote a healthy gut. In the event of inflammation such as in IBD, the breakdown in the intestinal barrier by immune system function, the microbial dysbiosis, and the dysregulation of glycosylation all have a combined effect on the resolution of inflammation. While direct evidence linking gut microbiota to expressions of morphogen and PDPN in IBD is limited, there are several potential mechanisms through which the microbiota could influence PDPN and morphogen levels as well as function. The relationship appears to be complex and bidirectional, with changes in microbiota affecting inflammation and lymphatic function, which, in turn, influence PDPN expression.

This review underscores the pivotal role of gut microbiota in the pathogenesis and management of IBD, presenting new opportunities for future research. By consolidating existing evidence, it provides a comprehensive framework to guide the exploration of microbiome-based diagnostic tools and therapies. Future studies can leverage the insights shared in this review to identify novel microbial biomarkers that more accurately predict disease progression, treatment response, or risk of complications. These findings could serve as the basis for developing precision medicine approaches that cater to the unique microbial compositions of individual patients.

Additionally, the mechanistic insights discussed, such as the interactions between gut microbiota and morphogens, glycosylation pathways, and podoplanin expression, provide a fertile ground for hypothesis-driven research. For instance, studying how microbial interventions influence these pathways could lead to groundbreaking therapeutic strategies aimed at restoring intestinal homeostasis. Furthermore, the role of dietary components, including iron, potassium, and calcium, on microbial diversity and gut health invites exploration into diet-–microbiome interactions and their implications for managing IBD symptoms and long-term outcomes.

Microbiota-based therapies for inflammatory bowel disease (IBD) offer several advantages, including targeted modulation of gut microbiota, disease-specific treatment approaches, and the continuous delivery of therapeutic compounds. Engineered bacteria can be designed to produce and release anti-inflammatory cytokines or metabolic products directly at the site of action, enhancing treatment efficacy. However, despite these benefits, several concerns must be considered. Potential risks include dependency on host immunity, the possibility of uncontrolled mutations, challenges related to microbial stability and colonization, the risk of horizontal gene transfer, and potential carcinogenic effects. Addressing these safety concerns is essential for the successful clinical implementation of microbiota-based therapies.

This review also highlights gaps in the current understanding of microbiota-host interactions, urging future investigations to employ advanced multi-omics techniques and longitudinal designs. These approaches could help delineate causal relationships and uncover hidden microbial signatures of therapeutic relevance. By building on the findings and perspectives discussed here, researchers can advance the field of gastroenterology, potentially transforming IBD treatment paradigms and improving patient quality of life.

Further research is needed to elucidate the specific pathways and interactions between morphogen and PDPN in gut microbiota in the context of IBD. In this review, we summarized various current associations between IBD and the gut microbiome. THe published literature has confirmed the effects of iron, potassium, and calcium on the gut microbiota and their subsequent impact on patients with IBD. We discussed how microbiota-focused pathways and proteins could be essential in creating treatments for human IBD. Ultimately, more research will be necessary to understand the connections between the host and microbes that are relevant to human disease and can be targeted for interventions.

## Figures and Tables

**Table 1:** Characteristics of IBD vs. healthy gut microbiota composition.

Reference	CD/UC/Both	Age	Sample	Microbe	Notes
[2]	CD	Adult	Resected ileum (paraffin blocks)	Bacterial	Some patients on antibiotics may have affected results
[3]	CD	Adult	Fecal	Bacterial	
[4]	CD	Adult	Fecal	Bacterial	
[5]	CD	Adult	Fecal, mucosal biopsy	Bacterial	
[6]	CD	Adult	Rectal mucosal biopsy	Bacterial	Compared healthy siblings
[7]	CD	Adult	Fecal	Bacterial	Compared healthy relatives
[8]	CD	Adult	Fecal	Bacterial	
[9]	CD	Adult	Fecal, mucosal biopsy	Bacterial	
[10]	CD	Adult	Colonic mucosal biopsy	Bacterial, fungal	
[11]	CD	Adult	Fecal	Bacterial	
[12]	CD	Adult	Fecal	Bacterial	
[13]	CD	Adult	Fecal	Bacterial	
[14]	CD	Adult *	Fecal	Bacterial	Compared healthy siblings
[15]	CD	Peds	Fecal	Bacterial	
[16]	CD	Peds	Ileal mucosal biopsy	Bacterial	
[17]	CD	Peds	Fecal	Bacterial	
[20]	UC	Adult	Fecal	Fungal	
[21]	UC	Adult	Fecal	Bacterial	Compared healthy relatives
[22]	Both CD/UC	Adult	Fecal	Bacterial	
[23]	Both CD/UC	Adult	Fecal	Bacterial	
[24]	Both CD/UC	Adult	Fecal	Bacterial	
[25]	Both CD/UC	Adult	Fecal	Bacterial	
[26]	Both CD/UC	Adult	Glucose hydrogen breath test	Bacterial	
[27]	Both CD/UC	Adult	Fecal	Bacterial	
[28]	Both CD/UC	Adult	Colonic mucosal biopsy	Bacterial	
[29]	Both CD/UC	Adult	Fecal	Bacterial	
[30]	Both CD/UC	Adult	Fecal	Bacterial	
[31]	Both CD/UC	Adult	Fecal	Bacterial	
[32]	Both CD/UC	Adult	Fecal	Bacterial	
[33]	Both CD/UC	Adult	Colonic mucosal biopsy	Bacterial	
[34]	Both CD/UC	Adult	Fecal	Bacterial	Compared healthy relatives
[35]	Both CD/UC	Adult	Fecal	Bacterial	
[36]	Both CD/UC	Adult	Fecal	Bacterial	
[37]	Both CD/UC	Adult	Fecal	Fungal	
[38]	Both CD/UC	Adult	Mucosal biopsy	Bacterial	
[39]	Both CD/UC	Adult	Fecal, mucosal biopsy	Bacterial	
[40]	Both CD/UC	Adult	Fecal	Bacterial	
[41]	Both CD/UC	Peds	Fecal	Bacterial	
[42]	Both CD/UC	Peds	Fecal	Viral	
[43]	Both CD/UC	Peds	Fecal	Bacterial	Compared healthy siblings
[44]	Both CD/UC	Peds	Fecal	Bacterial	

* Youngest patient 16 years old. CD: Crohn’s disease, UC: ulcerative colitis.

**Table 2:** IBD vs. healthy gut microbiota composition results summary.

Microbe	Disease and Reference
BACTERIAL	
Decreased *Bacteroidetes*	CD: [2,9]
	IBD: [35]
Decreased *Rikenellaceae*	CD: [8]
Decrease *Bacteroides*	IBD: [36]
Increase in *Bacteroidetes*	CD: [3] IBD: [28,31]
Increased *Bacteroides* (genus)	CD: [4,32]
	UC: [33]
	IBD: [38]
Increase *Bacteroides fragilis*	UC: [27]
Abundant *Firmicutes/Bacillota* (phylum)	CD: [2]
Increase in *Ruminococcus gnavus*	CD: [7]
Increased *Ruminococcus torques*	CD: [7]
Increased *Enterococcus* sp.	CD: [13,15,17,36]
	IBD: [23,24,40]
Increase *Faecalibacterium prausnitzii* species	CD: [16]
Increase *Veillonella parvula*	CD: [22]
Increased unclassified *Clostridium* genus	IBD: [41]
Increase *Strep mutans*	IBD: [41]
Increased *Lactobacillus*	CD: [30]
	UC: [33]
	IBD: [39]
Reduction in *Firmicutes/Bacillota* (phylum)	CD: [3,9,11,28]
	UC: [31]
	IBD: [27,30,35]
Decreased *Faecalibacterium prausnitzii*	CD: [6,7,9,11,14,15,17,22,34]
	UC: [21,43]
	IBD: [38,39]
Reductions in *Ruminococcaceae*	CD: [8]
	IBD: [44]
Decrease *Ruminococcus bromii*	CD: [15]
	UC: [27]
Reduced *Christensenellaceae*	CD: [8]
Decrease *Streptococcus gallolyticus*	CD: [9]
Decreased *Clostridia*	CD: [2,3,4]
	IBD: [32]
Decrease uncharacterized species of *Clostridium* cluster XIVa	CD: [7]
Decrease *Clostridia* cluster IV	CD: [14]
Decrease *Clostridium coccoides*	CD: [11,33]
*Clostriudium leptum*	CD: [11,33]
	IBD: [38]
Decrease *Clostridium colinum*	UC: [29]
Decrease *Gemmiger formicilis*	CD: [15]
Decrease *Veillonellaceae (Dialister)*	CD: [15]
Decrease *Eubacterium hallii*	IBD: [27]
Decrease *Eubacterium rectale*	UC: [43]
Decrease *Lachnospiraceae*	IBD: [27,44]
Decrease *Phascolarctobacterium*	IBD: [29]
Decrease *Butyricicoccus pullicaecorum*	UC: [29]
Decreased *Enterococcus*	UC: [36]
Decrease *Dialister invisus*	CD: [7]
Decrease *Lactobacillus coleohominis*	CD: [9]
Decrease *Roseburia* spp.	CD: [14,15,17]
	IBD: [29]
Decrease *Actinobacteria*	CD: [28,31]
Decreased *Bifidobacterium*	CD: [4,12]
	IBD: [36,44]
Decrease Bifidobacterium adolescentis	CD: [7,15]
Decrease *Coriobacteriaceae*	IBD: [44]
Decrease *Collinsella aerofaciens*	CD: [7]
Increased *Bifidobacterium*	UC: [39]
Enrichment of environmental *Mycobacterium*	CD: [2]
Increased Proteobacteria	CD: [10]
Abundances of *Gammaproteobacteria*	CD: [5]
	IBD: [31]
Abundant *Escherichia fergusonii*	CD: [6]
Increased member of the *Escherichia coli-Shigella* group	CD: [7]
Increase *E coli*	CD: [9,13,22]
	UC: [33,43]
	IBD: [28,30,36]
Increase in *Enterobacteriaceae*	CD: [8]
	IBD: [23,24]
Decrease *H. pylori*	IBD: [36]
Abundance of *Fusobacteria*	CD: [5,10]
	IBD: [35,40]
Increase *Methanosphaera stadtmanae*	IBD: [25]
Decrease *Akkermansia muciniphila*	UC: [29]
Increase Verrucomicrobia	IBD: [35]
Increase *Akkermansia muciniphila*	IBD: [41]
Decreased Tenericutes	CD: [31]
Decreased Cyanobacteria	IBD: [35]
FUNGAL	
Decreased *Saccharomyces cerevisiae*	CD: [10]
	UC: [20]
	IBD: [37]
Increased Cystofilobasidiaceae family	CD: [10]
*Filobasidium uniguttulatum* species associated with non-inflamed mucosa	CD: [10]
Xylariales order associated with inflamed mucosa	CD: [10]
Increased *Candida glabrata* species	CD: [10]
Absent *Candida deformans*, *Candida kefier*, *Candida parapsilosis*, *Rhodotorula* and *Kluyreromyces genera*	UC: [20]
Increased proportion of *Candida albicans*	IBD: [37]
Increased Basidiomycota/Ascomycota ratio	IBD: [37]
VIRAL:	
Higher ratio of *Caudovirales* to *Microviridae*	IBD: [42]
Increased *Anelloviridae*	IBD: [42]

CD: Crohn’s disease, UC: ulcerative colitis, IBD: irritable bowel disease.

**Table 3:** This represents the summary profile of various pre-clinical and clinical studies of how gut microbiota affects bile acids in IBD.

Type of Study	Aspect	Take Away Point
Clinical	Impact of Dysbiosis on IBD	IBD patients show reduced microbial diversity, decreased Firmicutes, increased Proteobacteria, and impaired BA transformation, leading to higher levels of primary BAs and lower levels of secondary BAs [121].
Preclinical	ASBT Expression in IBD	Inflammation and colitis models show decreased expression of the apical sodium-dependent bile acid transporter (ASBT), leading to disrupted BA recirculation and increased fecal BA accumulation [122].
Preclinical	Study on BA Homeostasis	Antibiotics treatment in mice increased BA absorption and decreased BA synthesis due to attenuation of gut microbiota [119].
Preclinical	Effects in colitis models	Rodent models of colitis show increased fecal BA accumulation and decreased BA recycling, with reduced BA transporter expression in models like TNBS- and DSS-induced colitis [123,124].
Clinical	Microbiome in Pediatric IBD	Pediatric IBD patients exhibit significantly reduced potential for BA production in their microbiome [125].
Preclinical	BA Levels in UC Patients	Colectomy-treated UC patients have decreased levels of secondary BAs (DCA and LCA) and fewer genes for converting primary to secondary BAs [126].
Preclinical	Colitis-Associated Cancer (CAC)	CAC mouse models show reduced fecal BAs, decreased transformation of primary to secondary BAs, and downregulation of the gut-liver FXR-FGF15 axis [127].

**Table 4:** This represents the summary profile of various pre-clinical and clinical studies of how gut microbiota affects immunity in IBD.

Study Type	Subject(s)	Key Findings	References
Animal	Mice (Pathogen-free, Germ-free IL10(−/−))	PSA from Bacteroides fragilis induces anti-inflammatory Tregs, protecting against colitis through IL-10-producing T cells.	[5,8]
Animal	Mice (Pathogen-free, Germ-free IL10(−/−))	Akkermansia muciniphila improves gut barrier function and reduces inflammation via its outer membrane protein Amuc_1100.	[5,8]
Human	46 IBD patients, 20 controls	Significantly reduced levels of Akkermansia muciniphila in IBD patients compared to controls.	[5,8]
Animal	Mice (FUT2 non-secretor)	Altered glycosylation patterns lead to bacterial overgrowth and increased susceptibility to inflammation, mimicking human IBD conditions.	[1,10]
Animal	Mice (MUC2 glycosylation defective)	Defective MUC2 glycosylation leads to spontaneous colitis, providing a model for colitis-associated cancer.	[1,10]
Human	IBD patients	Altered O-glycosylation profiles in UC patients are associated with increased inflammation and are reversible.	[1,10]
Human	IBD patients (different FUT2 genotypes)	FUT2 non-secretor status linked to distinct gut microbiota composition and increased IBD susceptibility.	[1,10]
Human	IBD patients	Elevated levels of fecal calprotectin and myeloperoxidase correlate with higher neutrophil activity and disease severity.	[2,129]
Human	41 IBD patients, 20 controls	IBD patients show significantly lower diversity and abundance of gut microbiota compared to healthy controls.	[3,130]

## Data Availability

Information for this review was collected from various sources, including PubMed, Google Scholar, Scopus, Web of Science, and ClinicalTrials.gov.

## References

[1] Lloyd-Price J., Arze C., Ananthakrishnan A.N., Schirmer M., Avila-Pacheco J., Poon T.W., Andrews E., Ajami N.J., Bonham K.S., Brislawn C.J. (2019). Multi-Omics of the Gut Microbial Ecosystem in Inflammatory Bowel Diseases. Nature.

[2] Molodecky N.A., Kaplan G.G. (2010). Environmental Risk Factors for Inflammatory Bowel Disease. Gastroenterol. Hepatol..

[3] Sher M.E., Bank S., Greenberg R., Sardinha C.T., Weissman S., Bailey B., Gilliland R., Wexner S.D. (1999). The Influence of Cigarette Smoking on Cytokine Levels in Patients with Inflammatory Bowel Disease. Inflamm. Bowel Dis..

[4] Hildebrand H., Malmborg P., Askling J., Ekbom A., Montgomery S.M. (2008). Early-Life Exposures Associated with Antibiotic Use and Risk of Subsequent Crohn’s Disease. Scand. J. Gastroenterol..

[5] Moller F.T., Andersen V., Wohlfahrt J., Jess T. (2015). Familial Risk of Inflammatory Bowel Disease: A Population-Based Cohort Study 1977–2011. Am. J. Gastroenterol..

[6] Turpin W., Goethel A., Bedrani L., Croitoru M.K. (2018). Determinants of IBD Heritability: Genes, Bugs, and More. Inflamm. Bowel Dis..

[7] Qin J., Li R., Raes J., Arumugam M., Burgdorf K.S., Manichanh C., Nielsen T., Pons N., Levenez F., Yamada T. (2010). A Human Gut Microbial Gene Catalogue Established by Metagenomic Sequencing. Nature.

[8] Glassner K.L., Abraham B.P., Quigley E.M.M. (2020). The Microbiome and Inflammatory Bowel Disease. J. Allergy Clin. Immunol..

[9] Chelakkot C., Ghim J., Ryu S.H. (2018). Mechanisms Regulating Intestinal Barrier Integrity and Its Pathological Implications. Exp. Mol. Med..

[10] Kudelka M.R., Stowell S.R., Cummings R.D., Neish A.S. (2020). Intestinal Epithelial Glycosylation in Homeostasis and Gut Microbiota Interactions in IBD. Nat. Rev. Gastroenterol. Hepatol..

[11] Zhou L., Zhang M., Wang Y., Dorfman R.G., Liu H., Yu T., Chen X., Tang D., Xu L., Yin Y. (2018). *Faecalibacterium prausnitzii* Produces Butyrate to Maintain Th17/Treg Balance and to Ameliorate Colorectal Colitis by Inhibiting Histone Deacetylase 1. Inflamm. Bowel Dis..

[12] Zheng L., Wen X.L., Duan S.L. (2022). Role of metabolites derived from gut microbiota in inflammatory bowel disease. World J. Clin. Cases.

[13] Deleu S., Machiels K., Raes J., Verbeke K., Vermeire S. (2021). Short Chain Fatty Acids and Its Producing Organisms: An Overlooked Therapy for IBD?. eBioMedicine.

[14] Furusawa Y., Obata Y., Fukuda S., Endo T.A., Nakato G., Takahashi D., Nakanishi Y., Uetake C., Kato K., Kato T. (2013). Commensal Microbe-Derived Butyrate Induces the Differentiation of Colonic Regulatory T Cells. Nature.

[15] Rolhion N., Chassaing B., Nahori M.-A., De Bodt J., Moura A., Lecuit M., Dussurget O., Bérard M., Marzorati M., Fehlner-Peach H. (2019). A *Listeria monocytogenes* Bacteriocin Can Target the Commensal *Prevotella copri* and Modulate Intestinal Infection. Cell Host Microbe.

[16] Dore M.P., Rocchi C., Longo N.P., Scanu A.M., Vidili G., Padedda F., Pes G.M. (2020). Effect of Probiotic Use on Adverse Events in Adult Patients with Inflammatory Bowel Disease: A Retrospective Cohort Study. Probiotics Antimicrob. Proteins.

[17] Turner D., Ricciuto A., Lewis A., D’Amico F., Dhaliwal J., Griffiths A.M., Bettenworth D., Sandborn W.J., Sands B.E., Reinisch W. (2021). STRIDE-II: An Update on the Selecting Therapeutic Targets in Inflammatory Bowel Disease (STRIDE) Initiative of the International Organization for the Study of IBD (IOIBD): Determining Therapeutic Goals for Treat-to-Target Strategies in IBD. Gastroenterology.

[18] Tamilarasan A.G., Cunningham G., Irving P.M., Samaan M.A. (2019). Recent advances in monoclonal antibody therapy in IBD: Practical issues. Frontline Gastroenterol..

[19] Stojanov S., Berlec A. (2024). Smart bionanomaterials for treatment and diagnosis of inflammatory bowel disease. Nanotechnol. Rev..

[20] Pittayanon R., Lau J.T., Leontiadis G.I., Tse F., Yuan Y., Surette M., Moayyedi P. (2020). Differences in Gut Microbiota in Patients With vs. Without Inflammatory Bowel Diseases: A Systematic Review. Gastroenterology.

[21] Pedamallu C.S., Bhatt A.S., Bullman S., Fowler S., Freeman S.S., Durand J., Jung J., Duke F., Manzo V., Cai D. (2016). Metagenomic Characterization of Microbial Communities In Situ Within the Deeper Layers of the Ileum in Crohn’s Disease. Cell. Mol. Gastroenterol. Hepatol..

[22] Rojas-Feria M., Romero-García T., Fernández Caballero-Rico J.Á., Pastor Ramírez H., Avilés-Recio M., Castro-Fernandez M., Chueca Porcuna N., Romero-Gόmez M., García F., Grande L. (2018). Modulation of Faecal Metagenome in Crohn’s Disease: Role of microRNAs as Biomarkers. World J. Gastroenterol..

[23] Andoh A., Kuzuoka H., Tsujikawa T., Nakamura S., Hirai F., Suzuki Y., Matsui T., Fujiyama Y., Matsumoto T. (2012). Multicenter Analysis of Fecal Microbiota Profiles in Japanese Patients with Crohn’s Disease. J. Gastroenterol..

[24] Eun C.S., Kwak M.-J., Han D.S., Lee A.R., Park D.I., Yang S.-K., Kim Y.S., Kim J.F. (2016). Does the Intestinal Microbial Community of Korean Crohn’s Disease Patients Differ from That of Western Patients?. BMC Gastroenterol..

[25] Hedin C., Van Der Gast C.J., Rogers G.B., Cuthbertson L., McCartney S., Stagg A.J., Lindsay J.O., Whelan K. (2016). Siblings of Patients with Crohn’s Disease Exhibit a Biologically Relevant Dysbiosis in Mucosal Microbial Metacommunities. Gut.

[26] Joossens M., Huys G., Cnockaert M., De Preter V., Verbeke K., Rutgeerts P., Vandamme P., Vermeire S. (2011). Dysbiosis of the Faecal Microbiota in Patients with Crohn’s Disease and Their Unaffected Relatives. Gut.

[27] Kennedy N.A., Lamb C.A., Berry S.H., Walker A.W., Mansfield J., Parkes M., Simpkins R., Tremelling M., Nutland S., UK IBD Genetics Consortium (2018). The Impact of NOD2 Variants on Fecal Microbiota in Crohn’s Disease and Controls Without Gastrointestinal Disease. Inflamm. Bowel Dis..

[28] Li Q., Wang C., Tang C., Li N., Li J. (2012). Molecular-Phylogenetic Characterization of the Microbiota in Ulcerated and Non-Ulcerated Regions in the Patients with Crohn’s Disease. PLoS ONE.

[29] Liguori G., Lamas B., Richard M.L., Brandi G., Da Costa G., Hoffmann T.W., Di Simone M.P., Calabrese C., Poggioli G., Langella P. (2016). Fungal Dysbiosis in Mucosa-Associated Microbiota of Crohn’s Disease Patients. J. Crohns Colitis.

[30] Ramakrishna B., Jayakanthan P., Pugazhendhi S., Kabeerdoss J. (2015). Alterations of Mucosal Microbiota in the Colon of Patients with Inflammatory Bowel Disease Revealed by Real Time Polymerase Chain Reaction Amplification of 16S Ribosomal Ribonucleic Acid. Indian J. Med. Res..

[31] Shiga H., Kajiura T., Shinozaki J., Takagi S., Kinouchi Y., Takahashi S., Negoro K., Endo K., Kakuta Y., Suzuki M. (2012). Changes of Faecal Microbiota in Patients with Crohn’s Disease Treated with an Elemental Diet and Total Parenteral Nutrition. Dig. Liver Dis..

[32] Zhang J., Chen S.-L., Li L.-B. (2017). Correlation between Intestinal Flora and Serum Inflammatory Factors in Patients with Crohn’s Disease. Eur. Rev. Med. Pharmacol. Sci..

[33] Hedin C.R., McCarthy N.E., Louis P., Farquharson F.M., McCartney S., Taylor K., Prescott N.J., Murrells T., Stagg A.J., Whelan K. (2014). Altered Intestinal Microbiota and Blood T Cell Phenotype Are Shared by Patients with Crohn’s Disease and Their Unaffected Siblings. Gut.

[34] Kowalska-Duplaga K., Gosiewski T., Kapusta P., Sroka-Oleksiak A., Wędrychowicz A., Pieczarkowski S., Ludwig-Słomczyńska A.H., Wołkow P.P., Fyderek K. (2019). Differences in the Intestinal Microbiome of Healthy Children and Patients with Newly Diagnosed Crohn’s Disease. Sci. Rep..

[35] Assa A., Butcher J., Li J., Elkadri A., Sherman P.M., Muise A.M., Stintzi A., Mack D. (2016). Mucosa-Associated Ileal Microbiota in New-Onset Pediatric Crohn’s Disease. Inflamm. Bowel Dis..

[36] Wang Y., Gao X., Ghozlane A., Hu H., Li X., Xiao Y., Li D., Yu G., Zhang T. (2018). Characteristics of Faecal Microbiota in Paediatric Crohn’s Disease and Their Dynamic Changes During Infliximab Therapy. J. Crohns Colitis.

[37] Sharifinejad N., Mozhgani S.H., Bakhtiyari M., Mahmoudi E. (2021). Association of LRRK2 *Rs11564258* Single Nucleotide Polymorphisms with Type and Extent of Gastrointestinal Mycobiome in Ulcerative Colitis: A Case–Control Study. Gut Pathog..

[38] Varela E., Manichanh C., Gallart M., Torrejón A., Borruel N., Casellas F., Guarner F., Antolin M. (2013). Colonisation by *Faecalibacterium prausnitzii* and Maintenance of Clinical Remission in Patients with Ulcerative Colitis. Aliment. Pharmacol. Ther..

[39] Serrano-Gómez G., Mayorga L., Oyarzun I., Roca J., Borruel N., Casellas F., Varela E., Pozuelo M., Machiels K., Guarner F. (2021). Dysbiosis and Relapse-Related Microbiome in Inflammatory Bowel Disease: A Shotgun Metagenomic Approach. Comput. Struct. Biotechnol. J..

[40] Roche-Lima A., Carrasquillo-Carrión K., Gómez-Moreno R., Cruz J.M., Velázquez-Morales D.M., Rogozin I.B., Baerga-Ortiz A. (2018). The Presence of Genotoxic and/or Pro-Inflammatory Bacterial Genes in Gut Metagenomic Databases and Their Possible Link With Inflammatory Bowel Diseases. Front. Genet..

[41] Jang H.-M., Kim J.-K., Joo M.-K., Shin Y.-J., Lee C.K., Kim H.-J., Kim D.-H. (2021). Transplantation of Fecal Microbiota from Patients with Inflammatory Bowel Disease and Depression Alters Immune Response and Behavior in Recipient Mice. Sci. Rep..

[42] Blais Lecours P., Marsolais D., Cormier Y., Berberi M., Haché C., Bourdages R., Duchaine C. (2014). Increased Prevalence of *Methanosphaera Stadtmanae* in Inflammatory Bowel Diseases. PLoS ONE.

[43] Ghoshal U.C., Yadav A., Fatima B., Agrahari A.P., Misra A. (2022). Small Intestinal Bacterial Overgrowth in Patients with Inflammatory Bowel Disease: A Case-Control Study. Indian J. Gastroenterol..

[44] Vatn S., Carstens A., Kristoffersen A.B., Bergemalm D., Casén C., Moen A.E.F., Tannaes T.M., Lindstrøm J., Detlie T.E., Olbjørn C. (2020). the IBD-Character Consortium. Faecal Microbiota Signatures of IBD and Their Relation to Diagnosis, Disease Phenotype, Inflammation, Treatment Escalation and Anti-TNF Response in a European Multicentre Study (IBD-Character). Scand. J. Gastroenterol..

[45] Stojanov S., Berlec A., Štrukelj B. (2020). The Influence of Probiotics on the Firmicutes/Bacteroidetes Ratio in the Treatment of Obesity and Inflammatory Bowel disease. Microorganisms.

[46] Bajer L., Kverka M., Kostovcik M., Macinga P., Dvorak J., Stehlikova Z., Brezina J., Wohl P., Spicak J., Drastich P. (2017). Distinct Gut Microbiota Profiles in Patients with Primary Sclerosing Cholangitis and Ulcerative Colitis. World J. Gastroenterol..

[47] Duboc H., Rajca S., Rainteau D., Benarous D., Maubert M.-A., Quervain E., Thomas G., Barbu V., Humbert L., Despras G. (2013). Connecting Dysbiosis, Bile-Acid Dysmetabolism and Gut Inflammation in Inflammatory Bowel Diseases. Gut.

[48] Imhann F., Vich Vila A., Bonder M.J., Fu J., Gevers D., Visschedijk M.C., Spekhorst L.M., Alberts R., Franke L., Van Dullemen H.M. (2018). Interplay of Host Genetics and Gut Microbiota Underlying the Onset and Clinical Presentation of Inflammatory Bowel Disease. Gut.

[49] Andoh A., Imaeda H., Aomatsu T., Inatomi O., Bamba S., Sasaki M., Saito Y., Tsujikawa T., Fujiyama Y. (2011). Comparison of the Fecal Microbiota Profiles between Ulcerative Colitis and Crohn’s Disease Using Terminal Restriction Fragment Length Polymorphism Analysis. J. Gastroenterol..

[50] Rajca S., Grondin V., Louis E., Vernier-Massouille G., Grimaud J.-C., Bouhnik Y., Laharie D., Dupas J.-L., Pillant H., Picon L. (2014). Alterations in the Intestinal Microbiome (Dysbiosis) as a Predictor of Relapse After Infliximab Withdrawal in Crohn’s Disease. Inflamm. Bowel Dis..

[51] Pascal V., Pozuelo M., Borruel N., Casellas F., Campos D., Santiago A., Martinez X., Varela E., Sarrabayrouse G., Machiels K. (2017). A Microbial Signature for Crohn’s Disease. Gut.

[52] Santoru M.L., Piras C., Murgia A., Palmas V., Camboni T., Liggi S., Ibba I., Lai M.A., Orrù S., Blois S. (2017). Cross Sectional Evaluation of the Gut-Microbiome Metabolome Axis in an Italian Cohort of IBD Patients. Sci. Rep..

[53] Sha S., Xu B., Wang X., Zhang Y., Wang H., Kong X., Zhu H., Wu K. (2013). The Biodiversity and Composition of the Dominant Fecal Microbiota in Patients with Inflammatory Bowel Disease. Diagn. Microbiol. Infect. Dis..

[54] Sokol H., Leducq V., Aschard H., Pham H.-P., Jegou S., Landman C., Cohen D., Liguori G., Bourrier A., Nion-Larmurier I. (2017). Fungal Microbiota Dysbiosis in IBD. Gut.

[55] Vrakas S., Mountzouris K.C., Michalopoulos G., Karamanolis G., Papatheodoridis G., Tzathas C., Gazouli M. (2017). Intestinal Bacteria Composition and Translocation of Bacteria in Inflammatory Bowel Disease. PLoS ONE.

[56] Wang W., Chen L., Zhou R., Wang X., Song L., Huang S., Wang G., Xia B. (2014). Increased Proportions of *Bifidobacterium* and the *Lactobacillus* Group and Loss of Butyrate-Producing Bacteria in Inflammatory Bowel Disease. J. Clin. Microbiol..

[57] Zhou Y., Chen H., He H., Du Y., Hu J., Li Y., Li Y., Zhou Y., Wang H., Chen Y. (2016). Increased *Enterococcus faecalis* Infection Is Associated with Clinically Active Crohn Disease. Medicine.

[58] Malham M., Lilje B., Houen G., Winther K., Andersen P.S., Jakobsen C. (2019). The Microbiome Reflects Diagnosis and Predicts Disease Severity in Paediatric Onset Inflammatory Bowel Disease. Scand. J. Gastroenterol..

[59] Liang G., Conrad M.A., Kelsen J.R., Kessler L.R., Breton J., Albenberg L.G., Marakos S., Galgano A., Devas N., Erlichman J. (2020). Dynamics of the Stool Virome in Very Early-Onset Inflammatory Bowel Disease. J. Crohns Colitis.

[60] Knoll R.L., Forslund K., Kultima J.R., Meyer C.U., Kullmer U., Sunagawa S., Bork P., Gehring S. (2017). Gut Microbiota Differs between Children with Inflammatory Bowel Disease and Healthy Siblings in Taxonomic and Functional Composition: A Metagenomic Analysis. Am. J. Physiol.-Gastrointest. Liver Physiol..

[61] Maukonen J., Kolho K.-L., Paasela M., Honkanen J., Klemetti P., Vaarala O., Saarela M. (2015). Altered Fecal Microbiota in Paediatric Inflammatory Bowel Disease. J. Crohns Colitis.

[62] Driever W., Nüsslein-Volhard C. (1988). The Bicoid Protein Determines Position in the Drosophila Embryo in a Concentration-Dependent Manner. Cell.

[63] Kicheva A., Briscoe J. (2023). Control of Tissue Development by Morphogens. Annu. Rev. Cell Dev. Biol..

[64] Rogers K.W., Schier A.F. (2011). Morphogen Gradients: From Generation to Interpretation. Annu. Rev. Cell Dev. Biol..

[65] Ashe H.L., Briscoe J. (2006). The Interpretation of Morphogen Gradients. Development.

[66] Moparthi L., Koch S. (2019). Wnt signaling in intestinal inflammation. Differentiation.

[67] Bajaj A., Markandey M., Kedia S., Ahuja V. (2024). Gut Bacteriome in Inflammatory Bowel Disease: An Update on Recent Advances. Indian J. Gastroenterol..

[68] Sugihara K., Kamada N. (2024). Metabolic Network of the Gut Microbiota in Inflammatory Bowel Disease. Inflamm. Regen..

[69] Gheonea D.I., Popa P., Săftoiu A. (2023). Inflammatory Bowel Diseases. Pocket Guide to Advanced Endoscopy in Gastroenterology.

[70] Balestrieri P., Ribolsi M., Guarino M.P.L., Emerenziani S., Altomare A., Cicala M. (2020). Nutritional Aspects in Inflammatory Bowel Diseases. Nutrients.

[71] Martini E., Krug S.M., Siegmund B., Neurath M.F., Becker C. (2017). Mend Your Fences. Cell. Mol. Gastroenterol. Hepatol..

[72] Wlodarska M., Kostic A.D., Xavier R.J. (2015). An Integrative View of Microbiome-Host Interactions in Inflammatory Bowel Diseases. Cell Host Microbe.

[73] Rees W.D., Sly L.M., Steiner T.S. (2020). How Do Immune and Mesenchymal Cells Influence the Intestinal Epithelial Cell Compartment in Inflammatory Bowel Disease? Let’s Crosstalk about It!. J. Leukoc. Biol..

[74] Lovisa S., Genovese G., Danese S. (2019). Role of Epithelial-to-Mesenchymal Transition in Inflammatory Bowel Disease. J. Crohns Colitis.

[75] Beck P.L., Podolsky D.K. (1999). Growth Factors in Inflammatory Bowel Disease. Inflamm. Bowel Dis..

[76] Wu H., Xie S., Miao J., Li Y., Wang Z., Wang M., Yu Q. (2020). *Lactobacillus reuteri* maintains intestinal epithelial regeneration and repairs damaged intestinal mucosa. Gut Microbes.

[77] Xie J., Li L., Dai T., Qi X., Wang Y., Zheng T., Gao X., Zhang Y., Ai Y., Ma L. (2021). Short-Chain Fatty Acids Produced by Ruminococcaceae Mediate α-Linolenic Acid Promote Intestinal Stem Cells Proliferation. Mol. Nutr. Food Res..

[78] Zhang L., Ocansey D.K.W., Liu L., Olovo C.V., Zhang X., Qian H., Xu W., Mao F. (2021). Implications of Lymphatic Alterations in the Pathogenesis and Treatment of Inflammatory Bowel Disease. Biomed. Pharmacother..

[79] Zhang Z., Zhang N., Yu J., Xu W., Gao J., Lv X., Wen Z. (2022). The Role of Podoplanin in the Immune System and Inflammation. J. Inflamm. Res..

[80] Zheng J., Sun Q., Zhang J., Ng S.C. (2022). The Role of Gut Microbiome in Inflammatory Bowel Disease Diagnosis and Prognosis. United Eur. Gastroenterol. J..

[81] Gubatan J., Boye T.L., Temby M., Sojwal R.S., Holman D.R., Sinha S.R., Rogalla S.R., Nielsen O.H. (2022). Gut Microbiome in Inflammatory Bowel Disease: Role in Pathogenesis, Dietary Modulation, and Colitis-Associated Colon Cancer. Microorganisms.

[82] Guo X., Huang C., Xu J., Xu H., Liu L., Zhao H., Wang J., Huang W., Peng W., Chen Y. (2022). Gut Microbiota Is a Potential Biomarker in Inflammatory Bowel Disease. Front. Nutr..

[83] Khan I., Ullah N., Zha L., Bai Y., Khan A., Zhao T., Che T., Zhang C. (2019). Alteration of Gut Microbiota in Inflammatory Bowel Disease (IBD): Cause or Consequence? IBD Treatment Targeting the Gut Microbiome. Pathogens.

[84] Becker F., Potepalov S., Shehzahdi R., Bernas M., Witte M., Abreo F., Traylor J., Orr W.A., Tsunoda I., Alexander J.S. (2015). Downregulation of FoxC2 Increased Susceptibility to Experimental Colitis: Influence of Lymphatic Drainage Function?. Inflamm. Bowel Dis..

[85] Wang X., Zhao J., Qin L. (2017). VEGF-C Mediated Enhancement of Lymphatic Drainage Reduces Intestinal Inflammation by Regulating IL-9/IL-17 Balance and Improving Gut Microbiota in Experimental Chronic Colitis. Am. J. Transl. Res..

[86] Barnhoorn M.C., Hakuno S.K., Bruckner R.S., Rogler G., Hawinkels L.J.A.C., Scharl M. (2020). Stromal Cells in the Pathogenesis of Inflammatory Bowel Disease. J. Crohns Colitis.

[87] Kotlarz D. (2022). Mucus Sialylation Maintains the Peace in Intestinal Host Microbe Relations. Gastroenterology.

[88] Saldova R., Thomsson K.A., Wilkinson H., Chatterjee M., Singh A.K., Karlsson N.G., Knaus U.G. (2024). Characterization of Intestinal O-Glycome in Reactive Oxygen Species Deficiency. PLoS ONE.

[89] Ma X., Li M., Wang X., Qi G., Wei L., Zhang D. (2024). Sialylation in the Gut: From Mucosal Protection to Disease Pathogenesis. Carbohydr. Polym..

[90] Lübbers J., Rodríguez E., Van Kooyk Y. (2018). Modulation of Immune Tolerance via Siglec-Sialic Acid Interactions. Front. Immunol..

[91] Bartsch Y.C., Rahmöller J., Mertes M.M.M., Eiglmeier S., Lorenz F.K.M., Stoehr A.D., Braumann D., Lorenz A.K., Winkler A., Lilienthal G.-M. (2018). Sialylated Autoantigen-Reactive IgG Antibodies Attenuate Disease Development in Autoimmune Mouse Models of Lupus Nephritis and Rheumatoid Arthritis. Front. Immunol..

[92] Brazil J.C., Parkos C.A. (2022). Finding the Sweet Spot: Glycosylation Mediated Regulation of Intestinal Inflammation. Mucosal Immunol..

[93] Pham T.A.N., Clare S., Goulding D., Arasteh J.M., Stares M.D., Browne H.P., Keane J.A., Page A.J., Kumasaka N., Kane L. (2014). Epithelial IL-22RA1-Mediated Fucosylation Promotes Intestinal Colonization Resistance to an Opportunistic Pathogen. Cell Host Microbe.

[94] He R., Li Y., Han C., Lin R., Qian W., Hou X. (2019). L-Fucose Ameliorates DSS-Induced Acute Colitis via Inhibiting Macrophage M1 Polarization and Inhibiting NLRP3 Inflammasome and NF-kB Activation. Int. Immunopharmacol..

[95] Corfield A.P., Myerscough N., Bradfield N., Do Amaral Corfield C., Gough M., Clamp J.R., Durdey P., Warren B.F., Bartolo D.C.C., King K.R. (1996). Colonic Mucins in Ulcerative Colitis: Evidence for Loss of Sulfation. Glycoconj. J..

[96] Tsai H.H., Dwarakanath A.D., Hart C.A., Milton J.D., Rhodes J.M. (1995). Increased Faecal Mucin Sulphatase Activity in Ulcerative Colitis: A Potential Target for Treatment. Gut.

[97] Ulmer J.E., Vilén E.M., Namburi R.B., Benjdia A., Beneteau J., Malleron A., Bonnaffé D., Driguez P.-A., Descroix K., Lassalle G. (2014). Characterization of Glycosaminoglycan (GAG) Sulfatases from the Human Gut Symbiont Bacteroides Thetaiotaomicron Reveals the First GAG-Specific Bacterial Endosulfatase. J. Biol. Chem..

[98] Santana P.T., Rosas S.L.B., Ribeiro B.E., Marinho Y., De Souza H.S.P. (2022). Dysbiosis in Inflammatory Bowel Disease: Pathogenic Role and Potential Therapeutic Targets. Int. J. Mol. Sci..

[99] Ning L., Zhou Y.-L., Sun H., Zhang Y., Shen C., Wang Z., Xuan B., Zhao Y., Ma Y., Yan Y. (2023). Microbiome and Metabolome Features in Inflammatory Bowel Disease via Multi-Omics Integration Analyses across Cohorts. Nat. Commun..

[100] Huang Y.-L., Chassard C., Hausmann M., Von Itzstein M., Hennet T. (2015). Sialic Acid Catabolism Drives Intestinal Inflammation and Microbial Dysbiosis in Mice. Nat. Commun..

[101] Suzuki T., Takahashi T., Guo C.T., Hidari K.I., Miyamoto D., Goto H., Kawaoka Y., Suzuki Y. (2005). Sialidase activity of influenza A virus in an endocytic pathway enhances viral replication. J. Virol..

[102] Wang Y.H. (2020). Sialidases From *Clostridium perfringens* and Their Inhibitors. Front. Cell Infect. Microbiol..

[103] Savageau M.A. (1986). Proteins of *Escherichia coli* Come in Sizes That Are Multiples of 14 kDa: Domain Concepts and Evolutionary Implications. Proc. Natl. Acad. Sci. USA.

[104] Sultan S., El-Mowafy M., Elgaml A., Ahmed T.A.E., Hassan H., Mottawea W. (2021). Metabolic Influences of Gut Microbiota Dysbiosis on Inflammatory Bowel Disease. Front. Physiol..

[105] Peng B., Xue L., Yu Q., Zhong T. (2023). Ellagic Acid Alleviates TNBS-Induced Intestinal Barrier Dysfunction by Regulating Mucin Secretion and Maintaining Tight Junction Integrity in Rats. Int. J. Food Sci. Nutr..

[106] Recharla N., Geesala R., Shi X.-Z. (2023). Gut Microbial Metabolite Butyrate and Its Therapeutic Role in Inflammatory Bowel Disease: A Literature Review. Nutrients.

[107] Yao Y., Kim G., Shafer S., Chen Z., Kubo S., Ji Y., Luo J., Yang W., Perner S.P., Kanellopoulou C. (2022). Mucus Sialylation Determines Intestinal Host-Commensal Homeostasis. Cell.

[108] Buzun E., Hsu C.-Y., Sejane K., Oles R.E., Vasquez Ayala A., Loomis L.R., Zhao J., Rossitto L.-A., McGrosso D.M., Gonzalez D.J. (2024). A Bacterial Sialidase Mediates Early-Life Colonization by a Pioneering Gut Commensal. Cell Host Microbe.

[109] Arpaia N., Campbell C., Fan X., Dikiy S., van der Veeken J., deRoos P., Liu H., Cross J.R., Pfeffer K., Coffer P.J. (2013). Metabolites Produced by Commensal Bacteria Promote Peripheral Regulatory T-Cell Generation. Nature.

[110] Ferdinandusse S., Houten S.M. (2006). Peroxisomes and Bile Acid Biosynthesis. Biochim. Biophys. Acta BBA—Mol. Cell Res..

[111] Sharma B., Twelker K., Nguyen C., Ellis S., Bhatia N.D., Kuschner Z., Agriantonis A., Agriantonis G., Arnold M., Dave J. (2024). Bile Acids in Pancreatic Carcinogenesis. Metabolites.

[112] Chen I., Cassaro S. (2024). Physiology, Bile Acids. StatPearls.

[113] Di Ciaula A., Garruti G., Lunardi Baccetto R., Molina-Molina E., Bonfrate L., Wang D.Q.-H., Portincasa P. (2017). Bile Acid Physiology. Ann. Hepatol..

[114] Hofmann A.F. (1999). The Continuing Importance of Bile Acids in Liver and Intestinal Disease. Arch. Intern. Med..

[115] Jones B.V., Begley M., Hill C., Gahan C.G.M., Marchesi J.R. (2008). Functional and Comparative Metagenomic Analysis of Bile Salt Hydrolase Activity in the Human Gut Microbiome. Proc. Natl. Acad. Sci. USA.

[116] Ridlon J.M., Harris S.C., Bhowmik S., Kang D.-J., Hylemon P.B. (2016). Consequences of Bile Salt Biotransformations by Intestinal Bacteria. Gut Microbes.

[117] Maruyama T., Miyamoto Y., Nakamura T., Tamai Y., Okada H., Sugiyama E., Nakamura T., Itadani H., Tanaka K. (2002). Identification of Membrane-Type Receptor for Bile Acids (M-BAR). Biochem. Biophys. Res. Commun..

[118] McGlone E.R., Bloom S.R. (2019). Bile Acids and the Metabolic Syndrome. Ann. Clin. Biochem. Int. J. Lab. Med..

[119] Out C., Patankar J.V., Doktorova M., Boesjes M., Bos T., De Boer S., Havinga R., Wolters H., Boverhof R., Van Dijk T.H. (2015). Gut Microbiota Inhibit Asbt-Dependent Intestinal Bile Acid Reabsorption via Gata4. J. Hepatol..

[120] Abraham C., Cho J.H. (2009). Inflammatory Bowel Disease. N. Engl. J. Med..

[121] Yang M., Gu Y., Li L., Liu T., Song X., Sun Y., Cao X., Wang B., Jiang K., Cao H. (2021). Bile Acid–Gut Microbiota Axis in Inflammatory Bowel Disease: From Bench to Bedside. Nutrients.

[122] Fitzpatrick L.R., Jenabzadeh P. (2020). IBD and Bile Acid Absorption: Focus on Pre-Clinical and Clinical Observations. Front. Physiol..

[123] Jahnel J., Fickert P., Hauer A.C., Högenauer C., Avian A., Trauner M. (2014). Inflammatory Bowel Disease Alters Intestinal Bile Acid Transporter Expression. Drug Metab. Dispos..

[124] Araki Y., Andoh A., Tsujikawa T., Fujiyama Y., Bamba T. (2001). Alterations in Intestinal Microflora, Faecal Bile Acids and Short Chain Fatty Acids in Dextran Sulphate Sodium-Induced Experimental Acute Colitis in Rats. Eur. J. Gastroenterol. Hepatol..

[125] Heinken A., Ravcheev D.A., Baldini F., Heirendt L., Fleming R.M.T., Thiele I. (2019). Systematic Assessment of Secondary Bile Acid Metabolism in Gut Microbes Reveals Distinct Metabolic Capabilities in Inflammatory Bowel Disease. Microbiome.

[126] Sinha S.R., Haileselassie Y., Nguyen L.P., Tropini C., Wang M., Becker L.S., Sim D., Jarr K., Spear E.T., Singh G. (2020). Dysbiosis-Induced Secondary Bile Acid Deficiency Promotes Intestinal Inflammation. Cell Host Microbe.

[127] Liu L., Yang M., Dong W., Liu T., Song X., Gu Y., Wang S., Liu Y., Abla Z., Qiao X. (2021). Gut Dysbiosis and Abnormal Bile Acid Metabolism in Colitis-Associated Cancer. Gastroenterol. Res. Pract..

[128] Chen F., Ma L., Sartor R.B., Li F., Xiong H., Sun A., Shneider B. (2002). Inflammatory-Mediated Repression of the Rat Ileal Sodium-Dependent Bile Acid Transporter by c-Fos Nuclear Translocation. Gastroenterology.

[129] Danne C., Skerniskyte J., Marteyn B., Sokol H. (2024). Neutrophils: From IBD to the Gut Microbiota. Nat. Rev. Gastroenterol. Hepatol..

[130] Schirmer M., Garner A., Vlamakis H., Xavier R.J. (2019). Microbial Genes and Pathways in Inflammatory Bowel Disease. Nat. Rev. Microbiol..

[131] Shan Y., Lee M., Chang E.B. (2022). The Gut Microbiome and Inflammatory Bowel Diseases. Annu. Rev. Med..

[132] De Musis C., Granata L., Dallio M., Miranda A., Gravina A.G., Romano M. (2020). Inflammatory Bowel Diseases: The Role of Gut Microbiota. Curr. Pharm. Des..

[133] Lauro M.L., Burch J.M., Grimes C.L. (2016). The Effect of NOD2 on the Microbiota in Crohn’s Disease. Curr. Opin. Biotechnol..

[134] Mottawea W., Chiang C.-K., Mühlbauer M., Starr A.E., Butcher J., Abujamel T., Deeke S.A., Brandel A., Zhou H., Shokralla S. (2016). Altered Intestinal Microbiota–Host Mitochondria Crosstalk in New Onset Crohn’s Disease. Nat. Commun..

[135] Graham D.B., Xavier R.J. (2020). Pathway Paradigms Revealed from the Genetics of Inflammatory Bowel Disease. Nature.

[136] Nagalingam N.A., Lynch S.V. (2012). Role of the Microbiota in Inflammatory Bowel Diseases. Inflamm. Bowel Dis..

[137] Ashton J.J., Boukas K., Stafford I.S., Cheng G., Haggarty R., Coelho T.A.F., Batra A., Afzal N.A., Williams A.P., Polak M.E. (2022). Deleterious Genetic Variation Across the NOD Signaling Pathway Is Associated With Reduced NFKB Signaling Transcription and Upregulation of Alternative Inflammatory Transcripts in Pediatric Inflammatory Bowel Disease. Inflamm. Bowel Dis..

[138] Candelli M., Franza L., Pignataro G., Ojetti V., Covino M., Piccioni A., Gasbarrini A., Franceschi F. (2021). Interaction between Lipopolysaccharide and Gut Microbiota in Inflammatory Bowel Diseases. Int. J. Mol. Sci..

[139] Caparrós E., Wiest R., Scharl M., Rogler G., Gutiérrez Casbas A., Yilmaz B., Wawrzyniak M., Francés R. (2021). Dysbiotic Microbiota Interactions in Crohn’s Disease. Gut Microbes.

[140] Gonçalves P., Araújo J.R., Di Santo J.P. (2018). A Cross-Talk Between Microbiota-Derived Short-Chain Fatty Acids and the Host Mucosal Immune System Regulates Intestinal Homeostasis and Inflammatory Bowel Disease. Inflamm. Bowel Dis..

[141] Cohen L.J., Cho J.H., Gevers D., Chu H. (2019). Genetic Factors and the Intestinal Microbiome Guide Development of Microbe-Based Therapies for Inflammatory Bowel Diseases. Gastroenterology.

[142] Bao M., Wang K., Li J., Li Y., Zhu H., Lu M., Zhang Y., Fan Q., Han L., Wang K. (2023). ROS Scavenging and Inflammation-Directed Polydopamine Nanoparticles Regulate Gut Immunity and Flora Therapy in Inflammatory Bowel Disease. Acta Biomater..

[143] Kunst C., Schmid S., Michalski M., Tümen D., Buttenschön J., Müller M., Gülow K. (2023). The Influence of Gut Microbiota on Oxidative Stress and the Immune System. Biomedicines.

[144] de Vos W.M., Tilg H., Van Hul M., Cani P.D. (2022). Gut Microbiome and Health: Mechanistic Insights. Gut.

[145] Sun Y., Wang X., Li L., Zhong C., Zhang Y., Yang X., Li M., Yang C. (2024). The Role of Gut Microbiota in Intestinal Disease: From an Oxidative Stress Perspective. Front. Microbiol..

[146] Kamiński M.M., Röth D., Krammer P.H., Gülow K. (2013). Mitochondria as Oxidative Signaling Organelles in T-Cell Activation: Physiological Role and Pathological Implications. Arch. Immunol. Ther. Exp..

[147] Davies M.J. (2005). The Oxidative Environment and Protein Damage. Biochim. Biophys. Acta.

[148] Dröge W. (2002). Free Radicals in the Physiological Control of Cell Function. Physiol. Rev..

[149] Aviello G., Knaus U.G. (2018). NADPH Oxidases and ROS Signaling in the Gastrointestinal Tract. Mucosal Immunol..

[150] Rogler G. (2017). Resolution of Inflammation in Inflammatory Bowel Disease. Lancet Gastroenterol. Hepatol..

[151] Pacher P., Beckman J.S., Liaudet L. (2007). Nitric Oxide and Peroxynitrite in Health and Disease. Physiol. Rev..

[152] Mantegazza A.R., Savina A., Vermeulen M., Pérez L., Geffner J., Hermine O., Rosenzweig S.D., Faure F., Amigorena S. (2008). NADPH Oxidase Controls Phagosomal pH and Antigen Cross-Presentation in Human Dendritic Cells. Blood.

[153] Kaminski M., Kiessling M., Süss D., Krammer P.H., Gülow K. (2007). Novel Role for Mitochondria: Protein Kinase Ctheta-Dependent Oxidative Signaling Organelles in Activation-Induced T-Cell Death. Mol. Cell. Biol..

[154] Kamiński M.M., Röth D., Sass S., Sauer S.W., Krammer P.H., Gülow K. (2012). Manganese Superoxide Dismutase: A Regulator of T Cell Activation-Induced Oxidative Signaling and Cell Death. Biochim. Biophys. Acta.

[155] Devadas S., Zaritskaya L., Rhee S.G., Oberley L., Williams M.S. (2002). Discrete Generation of Superoxide and Hydrogen Peroxide by T Cell Receptor Stimulation: Selective Regulation of Mitogen-Activated Protein Kinase Activation and Fas Ligand Expression. J. Exp. Med..

[156] Burova E., Borodkina A., Shatrova A., Nikolsky N. (2013). Sublethal Oxidative Stress Induces the Premature Senescence of Human Mesenchymal Stem Cells Derived from Endometrium. Oxid. Med. Cell. Longev..

[157] Babbs C.F. (1992). Oxygen Radicals in Ulcerative Colitis. Free Radic. Biol. Med..

[158] Singh V., Ahlawat S., Mohan H., Gill S.S., Sharma K.K. (2022). Balancing Reactive Oxygen Species Generation by Rebooting Gut Microbiota. J. Appl. Microbiol..

[159] Xue J., Ajuwon K.M., Fang R. (2020). Mechanistic Insight into the Gut Microbiome and Its Interaction with Host Immunity and Inflammation. Anim. Nutr..

[160] Wang Y., Wu Y., Wang Y., Xu H., Mei X., Yu D., Wang Y., Li W. (2017). Antioxidant Properties of Probiotic Bacteria. Nutrients.

[161] Feng T., Wang J. (2020). Oxidative Stress Tolerance and Antioxidant Capacity of Lactic Acid Bacteria as Probiotic: A Systematic Review. Gut Microbes.

[162] Shandilya S., Kumar S., Kumar Jha N., Kumar Kesari K., Ruokolainen J. (2022). Interplay of Gut Microbiota and Oxidative Stress: Perspective on Neurodegeneration and Neuroprotection. J. Adv. Res..

[163] González-Bosch C., Boorman E., Zunszain P.A., Mann G.E. (2021). Short-Chain Fatty Acids as Modulators of Redox Signaling in Health and Disease. Redox Biol..

[164] Lin S., Li Y., Zamyatnin A.A., Werner J., Bazhin A.V. (2018). Reactive Oxygen Species and Colorectal Cancer. J. Cell. Physiol..

[165] Hu Y., Chen D., Zheng P., Yu J., He J., Mao X., Yu B. (2019). The Bidirectional Interactions between Resveratrol and Gut Microbiota: An Insight into Oxidative Stress and Inflammatory Bowel Disease Therapy. BioMed Res. Int..

[166] Li L., Peng P., Ding N., Jia W., Huang C., Tang Y. (2023). Oxidative Stress, Inflammation, Gut Dysbiosis: What Can Polyphenols Do in Inflammatory Bowel Disease?. Antioxidants.

[167] Yin J., Ren W., Wei B., Huang H., Li M., Wu X., Wang A., Xiao Z., Shen J., Zhao Y. (2020). Characterization of Chemical Composition and Prebiotic Effect of a Dietary Medicinal Plant Penthorum Chinense Pursh. Food Chem..

[168] Stojanov S., Ravnikar M., Berlec A., Kreft S. (2021). Interaction between Silver Fir (*Abies alba*) Wood Water Extract and Lactobacilli. Die Pharm.-Int. J. Pharm. Sci..

[169] Yang W., Huang Z., Xiong H., Wang J., Zhang H., Guo F., Wang C., Sun Y. (2022). Rice Protein Peptides Alleviate Dextran Sulfate Sodium-Induced Colitis via the Keap1–Nrf2 Signaling Pathway and Regulating Gut Microbiota. J. Agric. Food Chem..

[170] Vaghari-Tabari M., Alemi F., Zokaei M., Moein S., Qujeq D., Yousefi B., Farzami P., Hosseininasab S.S. (2024). Polyphenols and Inflammatory Bowel Disease: Natural Products with Therapeutic Effects?. Crit. Rev. Food Sci. Nutr..

[171] Talero E., Avila-Roman J., Motilva V. (2012). Chemoprevention with Phytonutrients and Microalgae Products in Chronic Inflammation and Colon Cancer. Curr. Pharm. Des..

[172] Dong Y., Hou Q., Lei J., Wolf P.G., Ayansola H., Zhang B. (2020). Quercetin Alleviates Intestinal Oxidative Damage Induced by H_2_O_2_ via Modulation of GSH: In Vitro Screening and In Vivo Evaluation in a Colitis Model of Mice. ACS Omega.

[173] Malhotra A., Bath S., Elbarbry F. (2015). An Organ System Approach to Explore the Antioxidative, Anti-Inflammatory, and Cytoprotective Actions of Resveratrol. Oxid. Med. Cell. Longev..

